# Self-generating oxygen enhanced mitochondrion-targeted photodynamic therapy for tumor treatment with hypoxia scavenging

**DOI:** 10.7150/thno.36988

**Published:** 2019-09-20

**Authors:** Zhengyang Yang, Jiafeng Wang, Shichao Ai, Jianfei Sun, Xiaoli Mai, Wenxian Guan

**Affiliations:** 1 Department of General Surgery, Nanjing Drum Tower Hospital, The Affiliated Hospital of Nanjing University Medical School, No. 321 Zhongshan Road, Nanjing, 210008, China.; 2 School of Biological Science and Medical Engineering, Southeast University, No. 87 Dingjiaqiao, Nanjing, 210009, China; 3 Department of Radiology, Nanjing Drum Tower Hospital, The Affiliated Hospital of Nanjing University Medical School, No. 321 Zhongshan Road, Nanjing, 210008, China.

**Keywords:** mitochondrion targeting, endogenous oxygen generation, photodynamic therapy, tumor hypoxia, NIR fluorescence imaging.

## Abstract

Tumor hypoxia is an important reason for the limited therapeutic efficacy of photodynamic therapy (PDT) because of the oxygen requirement of the therapeutic process. PDT consumes tissue oxygen and destroys tumor vasculature, further hampering its own efficacy in promoting tumor deterioration. Therefore, overcoming the photodynamic exacerbation of tumor hypoxia is urgent.

**Methods:** Herein, we report a photodynamic nanoparticle with sustainable hypoxia remission skills by both intratumoral H_2_O_2_ catalysis and targeted mitochondrial destruction. The Mn_3_O_4_@MSNs@IR780 nanoparticles are formed by absorbing a photosensitizer (IR780) into 90 nm mesoporous silica nanoparticles (MSNs) and capping the surface pores with 5 nm Mn_3_O_4_ nanoparticles.

**Results:** These Mn_3_O_4_ nanoparticles can accumulate in tumors and respond to the H_2_O_2_-enriched tumor microenvironment by decomposing and catalyzing H_2_O_2_ into O_2_. Afterwards, IR780 is released and activated, spontaneously targeting the mitochondria due to its natural mitochondrial affinity. Under laser irradiation, this self-generated oxygen-enhanced PDT can destroy mitochondria and inhibit cell respiration, resulting in sustainable hypoxia remission in tumor tissues and consequently enhancing the therapeutic outcome. *In vitro* experiments suggest that Mn_3_O_4_@MSNs@IR780 exhibited highly mitochondrion-targeted properties and could sustainably inhibit tumor hypoxia. Additionally, the highest photoacoustic signal of HbO_2_ with the lowest Hb was observed in tumors from mice after PDT, indicating that these nanoparticles can also prevent tumor hypoxia *in vivo*.

**Conclusion:** Taken together, our study indicated a new approach for overcoming the sustainable hypoxia limitation in traditional PDT by targeted oxygen supplementation and mitochondria destruction.

## Introduction

Hypoxia has been recognized as one of the hostile hallmarks of most solid tumors due to the increasing metabolic processes of the destructively proliferating carcinoma cells, which eventually leads to a scant oxygen supply in the tumor microenvironment 1, 2. Hypoxia is also recognized as a dangerous factor that can cause tumor metastasis and angiogenesis 3, 4. The insufficient oxygen in tumor tissues is one of the major obstacles in successful photodynamic therapy (PDT) 5-7. As photochemical reactions require oxygen, PDT efficacy decreases exponentially with the consumption of oxygen during therapy, thus requiring a constant supply of oxygen 8-13. Additionally, the tumor hypoxic microenvironment may be exacerbated by the sustained consumption of oxygen in PDT 14.

Thus far, there have been different strategies to overcome the limitation of hypoxia limitation and consequently to improve PDT efficacy, such as transporting additional oxygen by perfluorocarbon, providing hyperbaric oxygen by inhalation and catalyzing endogenous H_2_O_2_ to O_2_ 15-17. Among these methods, the in situ production of oxygen via the employment of nanomaterials as a catalyst is the most effective. For instance, MnO_2_ and its various nanocomposites have recently attracted much attention as bioactive materials that can regulate oxygen in tumor hypoxia by the decomposition of endogenic H_2_O_2_ 18-20. However, these approaches face the severe problem that tumor hypoxia cannot be inhibited sustainably because the uninterrupted and heightened respiration in tumors consumes oxygen through the mitochondria. Mitochondria, as indispensable organelles responsible for cell respiration, have recently been indicated to play key roles in various human diseases, particularly malignancies 21, 22. Sustained respiration through the mitochondria may worsen tumor hypoxia, and mitochondria are always considered target organelles when designing targeted cancer therapy 23, 24. Therefore, the design of nanocomposites that can selectively destroy mitochondria and sustainably produce O_2_ in hypoxic tumors is of paramount importance.

**Scheme 1:**
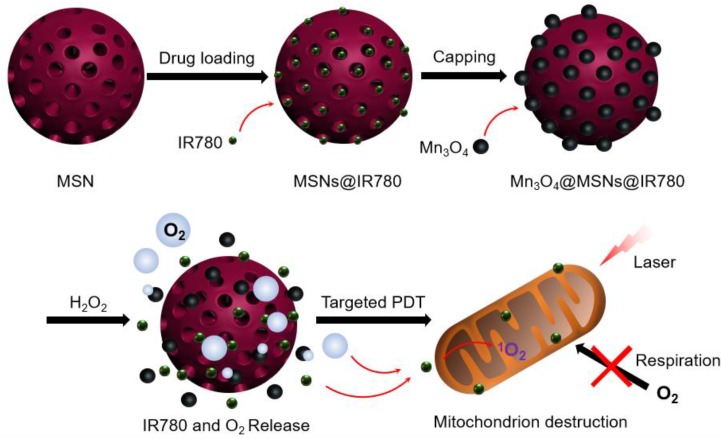
Schematic diagram of the synthetic processes of Mn_3_O_4_@MSNs@IR780, the H_2_O_2_ triggered release of IR780 and O_2_, and the mitochondria targeted PDT.

As a lipophilic cation, the near-infrared photosensitizer IR780 was found to accumulate predominantly in the mitochondria of tumor cells because of the higher mitochondrial membrane potential in tumor cells 25 (**Figure S1**). Mitochondrion-targeting PDT agents can rapidly damage the biological functions of the organelles under normal oxygen conditions, leading to the cell death of tumor cells. Moreover, the destruction of mitochondrial biological functions can inhibit cellular respiration within cancer cells, thus reducing oxygen consumption 26, 27. The vulnerability of mitochondria to reactive oxygen species (ROS) is a critical factor in designing a PDT system 28, 29. Based on these therapeutic approaches, in this work, a versatile nanocomposite (Mn_3_O_4_@MSNs@IR780) has been designed to concurrently achieve mitochondrion-targeted drug delivery, oxygen release, enhanced photodynamic therapy and sustained inhibition of hypoxia. Manganese oxide nanocrystals (Mn_3_O_4_ nanoparticles), which serve as gatekeepers, block the hydrophobic photosensitizer IR780-loaded channels of MSNs. H_2_O_2_ is one of the tumor metabolites (present at high amounts, up to 1 mM), and Mn_3_O_4_ nanoparticles in this study act as an efficient catalyst to continually break down H_2_O_2_ to oxygen without external activation 30-32. Moreover, the H_2_O_2_-responsive disintegration of Mn_3_O_4_ nanoparticles leads to oxygen generation and switch opening. Afterwards, IR780 dissociated and released in tumor tissues further specifically targets mitochondria because of its unique properties. Under the synergistic effect of oxygen and 808 nm laser irradiation, ROS are generated near mitochondria and damage them, further inhibiting cell respiration and leading to apoptosis of tumor cells (**Scheme 1**). The combined application of mitochondrial respiratory depression and self-generated oxygen may open a new path toward enlarging PDT curative effects.

## Experimental section

### Materials and instrumentation

Manganese acetate, N,N-dimethylformamide (DMF), 3-aminopropyltriethoxysilane (APTES), cetyltrimethylammonium bromide (CTAB), tetraethyl orthosilicate (TEOS, 99.98%), absolute ethanol, succinic anhydride, triethylamine, dimethyl sulfoxide (DMSO), 1-(3-(dimethylamino)propyl)-3-ethylcarbodiimide hydrochloride (EDC·HCl) and IR780 were purchased from Sigma-Aldrich. The Hydrogen Peroxide (H_2_O_2_) Colorimetric Assay Kit, Singlet Oxygen Sensor Green (SOSG), MitoTracker® Green FM, LysoTracker Green DND-26, the Reactive Oxygen Species Assay Kit, the ROS-ID™ Hypoxia/Oxidative Stress Detection Kit, the Membrane and Cytosol Protein Extraction Kit, PVDF membrane, Cell Counting Kit-8 and the Calcein-AM/PI Kit were purchased from Keygen Biotechnology.

The morphology of the nanocomposite was studied by transmission electron microscopy (TEM, FEI F20), high resolution TEM (JEOL, TEM-2100), field-emission scanning electron microscopy (SEM, JEOL) and dynamic light scattering (DLS, Nano-ZS90, Malvern, UK). The zeta potential of these nanoparticles was monitored by Zetaplus (Brookhaven Instruments Corporation). The fluorescence of IR780 and Mn_3_O_4_@MSNs@IR780 nanoparticles were detected using Fluoromax-4 spectrofluorometer (HoribaScientific, Edison, Nanjing). The stability of Mn_3_O_4_@MSNs@IR780 nanoparticles were monitored through their DLS and Zeta potential results every 12 h in PBS and serum. Structure characterization was performed via wide/small-angle X-ray diffraction (XRD) (Rigaku D/Ma 2550). Nitrogen adsorption-desorption isotherms were collected by an Autosorb iQ2 adsorptometer at 77 K. Fourier transform infrared (FTIR) spectroscopy (Nicolet Impact 410) and UV-vis-NIR spectroscopy (Shimadzu UV-3600) were used to determine the loading and release of IR780 or H_2_O_2_. The X-ray photoelectron spectroscopy (XPS) results were detected using an ESCALAB 250 spectrometer. A portable dissolved oxygen meter (YSI, 550A, Japan) was used to measure the production of oxygen.

### Synthesis of amine-functionalized Mn_3_O_4_

Mn_3_O_4_ nanoparticles were synthesized by the thermal decomposition of manganese acetate. Ten milliliters of manganese acetate was dissolved in 50 mL of DMF and loaded into a flask. Then, the temperature was raised to 130 ℃. After stabilization of the temperature, 500 µL of APTES was injected into the above solution, and a brown precipitate of amine-functionalized Mn_3_O_4_ nanoparticles was produced. The product was then centrifuged and washed three times with absolute ethanol.

### Synthesis of carboxyl-functionalized MSNs

Amine-functionalized MSNs of MCM-41 types (100 nm) were synthesized according to the following methods. First, 0.5 g CTAB (1.35 mmol) was dissolved in 240 mL of water. Then, 1.7 mL sodium hydroxide aqueous solution (2.00 M) was added to the CTAB solution, and the temperature of the mixture was raised to 80 °C. After achieving the desired temperature, 2.5 mL TEOS (11.2 mmol) and 250 μL APTES were successively added dropwise to the above alkaline surfactant solution under vigorous stirring. The mixture was stirred for 2 h to obtain a white precipitate. The resulting solid product was filtered, washed with water and ethanol, and dried at 60 °C. Next, 50 mg of MSNs-NH_2_ was further functionalized with carboxyl moieties using 5 mg of succinic anhydride and 5 µL of triethylamine in 10 mL of DMSO at 50 ℃ for 24 h.

### Preparation of MSNs@IR780

The photosensitizer IR780** (**ethanolic solution 5 mg/mL) was loaded into carboxyl-functionalized MSNs (50 mg) by sonication, followed by stirring for 10 h at room temperature. The green MSNs@IR780 powder was centrifuged and washed three times with ethanol and water. The loading amount of IR780 was determined by collection of the unloaded remaining solution and washings of IR780.

### Preparation of Mn_3_O_4_@MSNs@IR780

EDC chemistry was used to anchor Mn_3_O_4_ nanoparticles onto the surface of MSNs@IR780. Typically, 5 mg of EDC·HCl, 20 mg of MSNs@IR780, and 100 mg of Mn_3_O_4_ nanoparticles were dispersed into 5 mL of water, and the solution was stirred for 30 min. The Mn_3_O_4_@MSNs@IR780 nanocomposite was collected by centrifugation afterwards.

### Responsive IR780 release, oxygen production and PDT characterization

To analyze the catalytic ability of Mn_3_O_4_@MSNs@IR780 to generate oxygen from H_2_O_2_, a Hydrogen Peroxide (H_2_O_2_) Colorimetric Assay Kit (Elabscience Biotechnology, Wuhan, China) was used to determine the concentration of H_2_O_2_ over the course of investigation at 405 nm absorbance. The disintegration of Mn_3_O_4_ incubated in 1 mM H_2_O_2_ after 24 h was revealed through the appearance of the Mn 2p peak in the XPS spectrum. A portable dissolved oxygen meter was used to measure the real-time oxygen concentration with the oxygen electrode probe immersed in the solution after adding Mn_3_O_4_@MSNs@IR780 into 1 mM H_2_O_2_. The control release performance of IR780 was determined by measuring the absorbance of IR780 at 780 nm using UV-vis-NIR. The relevant pH-dependent and glutathione (GSH)-dependent decomposed behavior of Mn_3_O_4_@MSNs@IR780 were measured through the manganese (Mn^2+^) percentage concentration determined by ICP analysis.

The PDT characterization of Mn_3_O_4_@MSNs@IR780 was monitored using a ^1^O_2_-sensitive SOSG probe (Thermo Fisher Scientific, MA, USA). Briefly, 0.1 mL samples and 0.02 mL SOSG (50 μM) were added to 96-well plates without lighting. A multifunctional plate reader (Tecan Safire, Switzerland) was then used to measure the absorbance and fluorescence (λ_ex_ / λ_em_ = 488 nm / 520 nm) after 808 nm laser irradiation (1 W·cm^-2^, 5 min) under hermetically sealed conditions. The final IR780 concentration is 5 µg·mL^-1^.

### Cells and cell culture

The human gastric cancer cell line MKN-45P was obtained from the Shanghai Institute of Cell Biology, Chinese Academy of Sciences (Shanghai, China) and cultured in RPMI-1640 medium supplemented with 10% heat-inactivated FBS, 1% penicillin and 100 g·mL^-1^ streptomycin at 37 °C with 5% CO_2_.

### Subcellular localization of Mn_3_O_4_@MSNs@IR780

The subcellular localization of Mn_3_O_4_@MSNs@IR780 was detected by a confocal laser scanning microscope (Leica, Wetzlar, Germany). To demonstrate the mitochondrion targeting of IR780, mitochondrion- and lysosome-selective fluorescence probes were used as subcellular location marker. After 4 h of co-culture of MKN-45P cells with Mn_3_O_4_@MSNs@IR780 and Mn_3_O_4_@MSNs, MitoTracker® Green FM (λ_ex_ / λ_em_ = 490 nm / 516 nm) and LysoTracker Green DND-26 (λ_ex_ / λ_em_ = 504 nm / 511 nm) were added to label the mitochondria and lysosomes, respectively, for 45 min. After washing three times with PBS, the subcellular localization of Mn_3_O_4_@MSNs@IR780 and Mn_3_O_4_@MSNs was observed using confocal laser scanning microscopy (CLSM).

### ROS generation in cancer cells

ROS generation in cancer cells was detected using a Reactive Oxygen Species Assay Kit (λ_ex_ / λ_em_ = 488 nm / 525 nm). Six groups were established (control, laser, IR780, IR780 + laser, Mn_3_O_4_@MSNs@IR780 and Mn_3_O_4_@MSNs@IR780 + laser). All analyses were performed three times. MKN-45P cells were seeded and incubated on a 6-well plate (or in six confocal cell culture dishes) for 12 h. Then, 1 mL of RPMI-1640, 5 µg of IR780 dispersed in 1 mL of RPMI-1640, and Mn_3_O_4_@MSNs@IR780 dispersed in 1 mL of RPMI-1640 (5 µg IR780) were added to the corresponding wells (or dishes). Cells were incubated for another 4 h at 37 °C and then washed with PBS three times. Subsequently, 1 mL of DCFH‐DA was added to each well (500 µL to each dish) and incubated for another 15 min. Next, the cells were washed three times and irradiated with an 808 nm laser (1 W·cm^-2^, 5 min). The generation of ROS was then quantitatively measured using flow cytometry (Becton Dickinson Bioscience, San Jose, CA, USA). Meanwhile, the generation of ROS in MKN-45P cells was also observed using CLSM.

### Hypoxia detection in cancer cells

The ROS-ID™ Hypoxia/Oxidative Stress Detection Kit (Enzo Life Sciences) was used to evaluate the hypoxia conditions in cancer cells. Four groups were established (control, IR780 + laser, Mn_3_O_4_@MSNs@IR780 and Mn_3_O_4_@MSNs@IR780 + laser). In addition, we repeated the experiments 6 h after treatment to estimate the ability to sustain the prevention of hypoxia. The experimental methods were identical to those used for ROS detection experiments. Subsequently, the hypoxia/oxidative stress detection mixture was added to confocal cell culture dishes following the manufacturer's instructions. After incubation for 30 min, the MKN-45P cells were washed with PBS three times and irradiated with an 808 nm laser (1 W·cm^-2^, 5 min). Next, the ROS signal (λ_ex_ / λ_em_ = 488 nm / 520 nm) and the hypoxia signal (λ_ex_ / λ_em_ = 488 nm / 590 nm) were monitored using CLSM.

Western blot analysis was further conducted to detect the expression of hypoxia inducible factor-1α (HIF-1α) in gastric cancer cells. Total protein in MKN-45P cells was extracted using the Membrane and Cytosol Protein Extraction Kit (Beyotime Biotechnology). Samples were then transferred onto a PVDF membrane (Millipore, MA, USA). The membranes were then blocked with 5% skimmed milk and incubated with the primary antibody, anti-GAPDH and anti-HIF-1α, (Cell Signaling Technology) overnight at 8 °C. Anti-rabbit secondary antibodies were used, and bands were visualized by Pierce chemiluminescent substrate (Thermo Fisher). Photographs were acquired using FLI Capture (Tanon, Shanghai, China) and analyzed using ImageJ software.

### *In vitro* therapy against cancer cells

To assess the therapeutic effects of different treatments, MKN-45P cells were seeded in 96‐well plates for 12 h. The groups and experimental approaches were similar to those used for ROS generation detection. The final IR780 concentration was 5 µg·mL^-1^. After 4 h incubation with each sample, MKN-45P cells were washed three times with PBS and then exposed to a 808 nm laser (1 W·cm^-2^, 5 min per well). Next, 10 μL of Cell Counting Kit-8 (CCK-8) was added to the wells for a further 4 h incubation, and an ELISA microplate reader was used to measure the absorbance values in each well at 450 nm. Each group was examined six times.

Additionally, the Calcein-AM/PI Kit was used to identify living and dead MKN-45P cells. After the 808 nm laser (1 W·cm^-2^, 5 min) treatment, 200 μL of cancer cell suspension (10^5^ cells) was incubated with 100 μL of CAM/PI Double Stain working solution following the manufacturer's instructions. Subsequently, the cells were washed three times with PBS. Living cells (λ_ex_ / λ_em_ = 490 nm / 515 nm) and dead cells (λ_ex_ / λ_em_ = 535 nm / 617 nm) were monitored using CLSM. The counts of green/red cells were analyzed using ImageJ software.

### MKN-45P tumor xenograft model

Five-week-old male BALB/c nude mice with severe combined immunodeficiency (SCID) were obtained from the Model Animal Research Center of Nanjing University (Nanjing, China). All animal experiments were approved by the Institutional Animal Care and Use Committee (IACUC) of Nanjing University. When the MKN-45P cells reached 90% confluence, they were subcultured at a ratio of 1:3. To establish the MKN-45P tumor xenograft models, a total of 1 × 10^6^ MKN-45P cells were suspended in 100 μL of PBS, then injected subcutaneously into the left flank area of nude mice. The tumor volume was calculated as [π / 6 × length × (width)^2^].

### *In vivo* NIR fluorescence imaging and biodistribution

For *in vivo* NIR fluorescence imaging and biodistribution assessment, a Mn_3_O_4_@MSNs@IR780 PBS suspension was injected through the tail veins. The final IR780 concentration was 25 µg·kg^-1^ per mouse. These mice were then imaged by the Maestro *in vivo* fluorescence imaging system (Cri Inc., Woburn, MA) before and 0.5, 1, 2, 4, 6, 8, 12, 24, 48 h post injection. Subsequently, the mice were sacrificed, and the main organs and tumor tissues were imaged to detect the biodistribution of Mn_3_O_4_@MSNs@IR780 at 24 h. Meanwhile, MKN‐45P tumor xenograft mice were sacrificed 4, 12, 24, and 48 h after intravenous injection into the tail vein. Tumor tissues and major organs (heart, liver, spleen, lung and spleen) were collected, weighed, and lysed in aqua regia. The content of manganese was measured using a NexION 300D ICP‐MS.

### Monitoring tumor hypoxia conditions

To monitor the tumor hypoxia conditions, the xenograft mice were divided into six groups (control, laser, IR780, IR780 + laser, Mn_3_O_4_@MSNs@IR780 and Mn_3_O_4_@MSNs@IR780 + laser). The mice were injected through the tail veins with different agents (PBS, IR780 and Mn_3_O_4_@MSNs@IR780) and then split into laser and no laser subgroups. The final IR780 concentration was 25 µg·kg^-1^ per mouse. Then, photoacoustic imaging (PA) was performed to monitor the vascular saturated oxygen in tumor tissues 24 h after injection. Oxygenated hemoglobin (HbO_2_) was detected at an excitation wavelength of 850 nm and deoxygenated hemoglobin (Hb) at 700 nm using a preclinical photoacoustic computerized tomography scanner (Endra Nexus 128, USA) 24 h after injection. Subsequently, the mice were sacrificed, and tumor tissues were harvested for HIF‐1α immunohistochemical analysis. The PA intensity and counts of blue/brown cells were analyzed using ImageJ software.

### *In vivo* antitumor therapy

The tumor-bearing MKN-45P xenograft mice were randomly divided into six groups (control, laser, IR780, IR780 + laser, Mn_3_O_4_@MSNs@IR780 and Mn_3_O_4_@MSNs@IR780 + laser) when the tumor volume reached approximately 50 mm. The control group was injected with 100 µL of PBS per mouse, while the laser group was injected with 100 µL PBS and then irradiated with an 808 nm laser (1 W·cm^-2^, 5 min per mouse). The IR780 group was injected with 100 µL IR780 PBS suspension per mouse. The IR780 + laser group was injected with IR780 PBS suspension followed by 808 nm laser (1 W·cm^-2^, 5 min per mouse). The Mn_3_O_4_@MSNs@IR780 group was injected with Mn_3_O_4_@MSNs@IR780 PBS suspension per mouse. The Mn_3_O_4_@MSNs@IR780 + laser group was intravenously injected with the Mn_3_O_4_@MSNs@IR780 PBS suspension followed by an 808 nm laser (1 W·cm^-2^, 5 min per mouse). The final IR780 concentration was 25 µg·kg^-1^ per mouse. The body weight and tumor volume of each mouse were observed and recorded every two days after laser irradiation. After 16 days, the mice were sacrificed, and tumor tissues were collected, washed three times with PBS and weighed. Then, the tumor issues and major organs, including the heart, liver, spleen, lung and kidney, were harvested and fixed in a 4% paraformaldehyde solution. Finally, tumor issues were stained with hematoxylin and eosin (H & E) and TUNEL for histopathology analysis.

### Biosafety analysis of Mn_3_O_4_@MSNs@IR780

To observe the pathology changes, major organs (heart, liver, spleen, lung, and kidney) collected from the mice after therapy were fixed in a 4% paraformaldehyde solution at 4 °C for 4 h and then embedded in paraffin. Then, these tissues were stained with H & E, and the histopathologic changes were detected by an optical microscope (Olympus, Japan).

Hematological and biochemical assays were also used to evaluate the *in vivo* toxicity and biocompatibility of Mn_3_O_4_@MSNs@IR780. The mice were anesthetized, and blood was collected from the naked eye for hematological and biochemical assays, including white blood cell (WBC), neutrophil (NEU), lymphocyte (LYM), red blood cell (RBC), hemoglobin (HGB), platelet (PLT), alanine aminotransferase (ALT), aspartate aminotransferase (AST), blood urea nitrogen (BUN) and serum creatinine (Scr) assays.

### Statistical analysis

All data were analyzed using GraphPad Prism (version 5.01) software at a significance level of *p < 0.05; **p < 0.01 and ***p < 0.001. All data are presented as the mean ± standard deviation (SD).

## Results and Discussion

### Synthesis and characterizations of Mn_3_O_4_@MSNs@IR780

**Figure 1:**
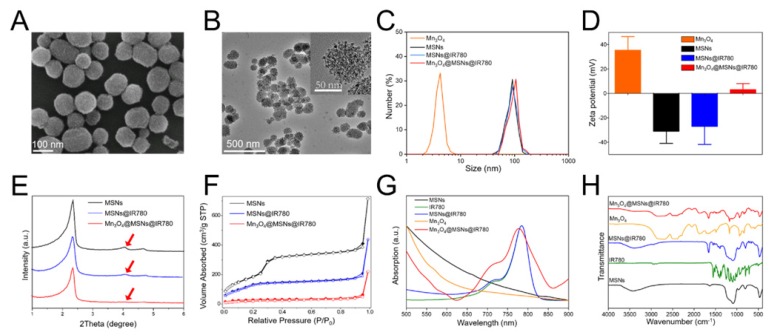
Characterizations of Mn_3_O_4_@MSNs@IR780 nanoparticles. (A) SEM image of Mn_3_O_4_@MSNs@IR780. (B) TEM images of Mn_3_O_4_@MSNs@IR780. The inset is a high resolution photo to clearly demonstrate the capping of MSNs by Mn_3_O_4_ nanoparticles. (C) Dynamic light scattering (DLS) of different MSNs and Mn_3_O_4_ nanoparticles. (D) Zeta potentials of different MSNs and Mn_3_O_4_ nanoparticles. (E) Small-angle powder XRD patterns of different MSNs nanoparticles. (F) Nitrogen adsorption-desorption isotherms of different MSNs nanoparticles. (G) UV-vis-NIR spectrums of IR780, different MSNs and Mn_3_O_4_ nanoparticles. (H) Fourier transform infrared (FTIR) spectrums of IR780, different MSNs and Mn_3_O_4_ nanoparticles (n = 3). Data is shown as mean ± SD.

MSN-based drug nanocarriers were first synthesized and then functionalized with amine moieties, thus improving their water stability and providing anchoring sites for gatekeepers. After IR780 was loaded into functionalized MSNs by sonication, Mn_3_O_4_ nanoparticles (5 nm) were anchored as gatekeepers on the IR780-loaded MSNs (MSNs@IR780) using EDC chemistry. Scanning electron microscopy (SEM) and transmission electron microscopy (TEM) images of Mn_3_O_4_@MSNs@IR780 showed the uniform distribution and considerable blockade of IR780 molecules by Mn_3_O_4_ nanoparticles to prevent IR780 leakage from the MSN nanochannels before reaching the targeted region (**Figure 1A-B**). TEM images of the Mn_3_O_4_ nanoparticles with an average particle diameter of 5 nm are provided in **Figure S2**.

The size of MSNs was approximately 88 nm, while the size of Mn_3_O_4_@MSNs@IR780 was approximately 94 nm (**Figure 1C**). Additionally, the polymer dispersity index (PDI) value of MSNs and Mn_3_O_4_@MSNs@IR780 was about 0.39 and 0.47, indicating that such nanoparticles exhibited well stability and dispersity. The zeta potential of Mn_3_O_4_, MSNs, MSNs@IR780 and Mn_3_O_4_@MSNs@IR780 was approximately 35 mV, -31 mV, -27 mV and 3 mV, which revealed the successful surface conjugation of Mn_3_O_4_ with MSNs@IR780 (**Figure 1D**). After drug loading and Mn_3_O_4_ conjugation, the typical MSN peaks (red arrow) decreased markedly in low-angle X-ray diffraction (XRD) results, demonstrating that holes on their surface might have already been covered by IR780 and Mn_3_O_4_ (**Figure 1E**). During drug loading and surface capping, the BET surface areas of MSNs, MSNs@IR780, and Mn_3_O_4_@MSNs@IR780 gradually decreased, as shown in the nitrogen adsorption analysis (**Figure 1F**). Well-ordered MSN nanopores can be seen in the high-resolution TEM micrograph (**Figure S3A**). According to the adsorption desorption isotherms, the corresponding pore size distributions were calculated for all three samples. As shown in **Figure S3B**, the pore size of MSNs possesses a narrow distribution at approximately 2.45 nm, while a wide distribution from 1.4 to 2.7 nm was found in the drug-loaded sample. Pore size distribution data of Mn3O4@MSNs@IR780 revealed blockage of the drug-loaded nanopores of MSNs. Meanwhile, both the UV-vis-NIR and Fourier transform infrared (FTIR) spectra of Mn_3_O_4_@MSNs@IR780 featured specific absorption peaks of IR780 and Mn_3_O_4_, thus implying the successful loading of the hydrophobic photosensitizer and the H_2_O_2_ response switch (**Figure 1G-H**).

The loading amount of IR780 in the MSNs was determined by UV-vis-NIR and found to be as high as 12 mg per 1 g of MSNs. As shown in **Figure S4**, we could observe the fluorescence of IR780 and Mn_3_O_4_@MSNs@IR780 nanoparticles was similar, indicating that fluorescence would not be quenched after loading to MSNs. DLS and Zeta potential of Mn_3_O_4_@MSNs@IR780 could maintain stable for 50 h in PBS and serum, demonstrating that these nanoparticles had a superior stability (**Figure S5**).

### *In vitro* H_2_O_2_ responsive drug release and catalytic/photodynamic effect

**Figure 2:**
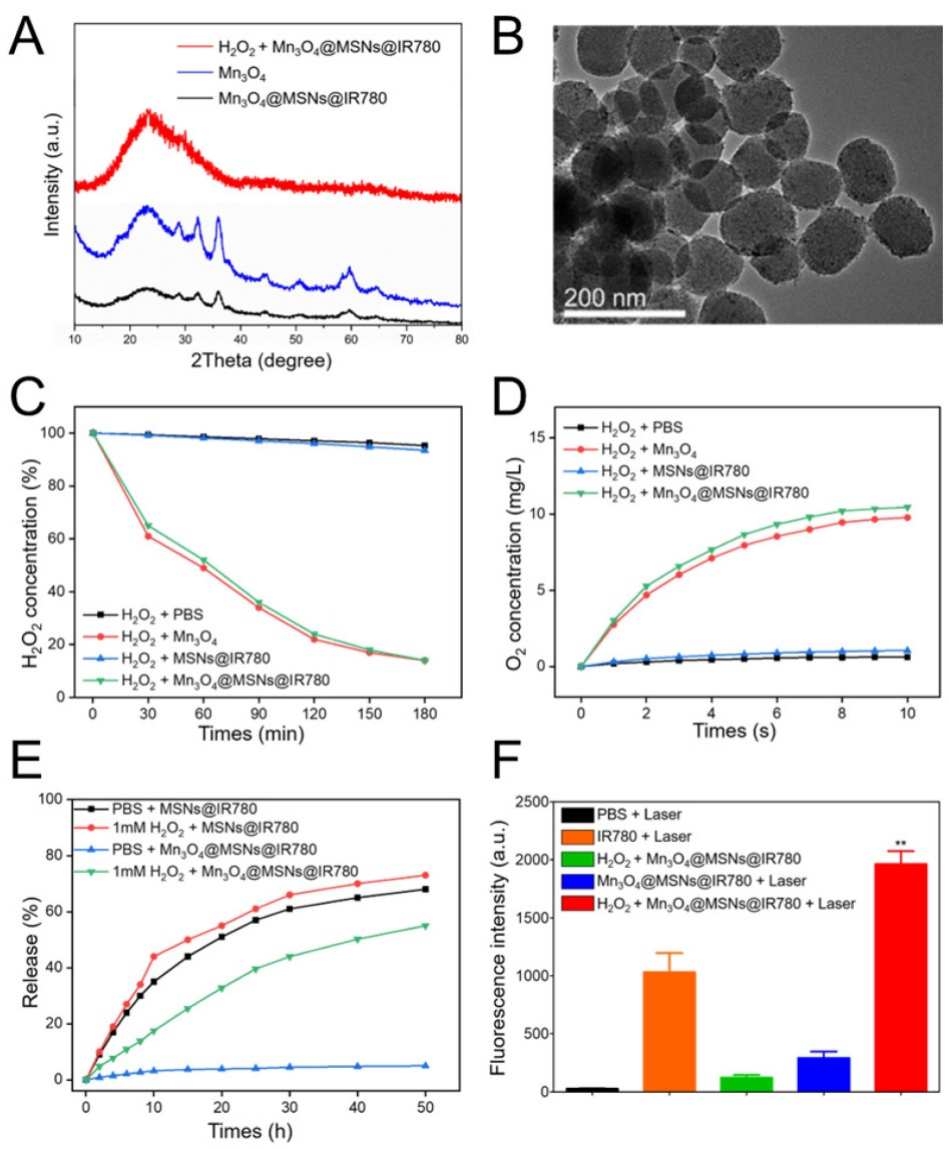
** Responsive drug release and further oxygen and ROS production from different samples. (A)** Wide-angle powder XRD patterns of Mn_3_O_4_@MSNs@IR780 nanoparticles incubated in 1 mM H_2_O_2_ after 24 h. **(B)** TEM image of Mn_3_O_4_@MSNs@IR780 nanoparticles incubated in 1 mM H_2_O_2_ after 24 h. **(C)** Degradation of H_2_O_2_ after reacting with different nanoparticles in H_2_O_2_ solution. **(D)** Generation of oxygen after reacting with different nanoparticles in H_2_O_2_ solution. **(E)** IR780 release profiles in different simulated conditions. **(F)** ROS generation in different simulated conditions (n = 3). Irradiation was performed by 808 nm laser (1 W·cm^-2^, 5min). Data is shown as mean ± SD, H_2_O_2_ + Mn_3_O_4_@MSNs@IR780 + Laser versus IR780 + laser, *p < 0.05; **p < 0.01 and ***p < 0.001.

Exposure to a specific high H_2_O_2_ concentration in the tumor microenvironment resulted in the steady-state dissolution of these Mn_3_O_4_ nanoparticles and the subsequent generation of oxygen 33,34. To verify the Mn_3_O_4_ response to H_2_O_2_, wide-angle powder XRD patterns were tested. The characteristic peaks of Mn_3_O_4_ decreased markedly in H_2_O_2_ solution, indicating the decomposition and abscission of Mn_3_O_4_ from the MSNs (**Figure 2A**). The nitrogen adsorption analysis pointed out the decomposition and abscission of Mn_3_O_4_ from Mn_3_O_4_@MSNs@IR780 after incubated in H_2_O_2_ for 24 h (**Figure S6**). As shown in **Figure S7A**, the characteristic peak of Mn_3_O_4_ at the wavenumber of about 2500 cm^-1^ disappeared after incubated in H_2_O_2_. The characteristic peak of IR780 also disappeared in **Figure S7B**, demonstrated that the opening of MSNs channels and further release of drugs. As for the XPS spectrum results, the counts of Mn 2p_3/2_ and Mn 2p_1/2_ at BE values of 641.7 eV and 653.7 eV decreased obviously while incubated in H_2_O_2_ for 24 h, also indicating the successfully decomposing of Mn_3_O_4_ after H_2_O_2_ treatment (**Figure S7C-F**).

Such results above demonstrated that the Mn_3_O_4_ nanoparticles were disintegrated and further opened the channels of MSNs. Before H_2_O_2_ treatment, the wide-angle XRD pattern of Mn_3_O_4_@MSNs@IR780 nanoparticles was well indexed to the tetragonal hausmannite structure (JCPDS 24-07340), while the XPS data showed that the BE values of Mn 2p_3/2_ and 2p_1/2_ were 641.7 eV and 653.7 eV, respectively. These results indicated the presence of dual oxidation states, Mn^2+^ and Mn^3+^, in the Mn_3_O_4_@MSNs@IR780 nanoparticles before reaction with H_2_O_2_. According to previous studies 35,36, Mn_3_O_4_ nanoparticles possess the catalase-mimicking ability to catalyze endogenous tumor H_2_O_2_ into O_2_, wherein H_2_O_2_ acted as the reducing agent and could reduce the trivalent Mn to divalent Mn and break itself down into H_2_O and O_2_. In our study, as expected, a transparent solution was observed after Mn_3_O_4_@MSNs@IR780 reacted with H_2_O_2_, indicating the transformation of trivalent Mn into divalent Mn after reaction with H_2_O_2_. The TEM image showed that the nanoparticles became smoother with fewer blackspots on their surface, which is further evidence of Mn_3_O_4_ decomposition and abscission from MSNs (**Figure 2B**).

To estimate the catalytic efficiency of Mn_3_O_4_@MSNs@IR780, the time-dependent decomposition of H_2_O_2_ was investigated in the presence of Mn_3_O_4_@MSNs@IR780. H_2_O_2_ was completely decomposed within 3 h (**Figure 2C**). In addition, single Mn_3_O_4_ nanoparticles exhibited similar H_2_O_2_ decomposition efficiencies. A portable dissolved oxygen meter was used to identify that the produced bubbles were oxygen. The real-time oxygen concentration increased rapidly after the addition of both Mn_3_O_4_ and Mn_3_O_4_@MSNs@IR780 into H_2_O_2_ solution, while MSNs@IR780 exhibited almost no oxygen-generation ability (**Figure 2D**). Meanwhile, we have also investigated the dissolution rate of Mn_3_O_4_ in various environments in the presence of 1 mM H_2_O_2_ and glutathione (GSH) to mimic acidic and oxidative stressed tumor conditions. After 24h exposure to acidic H_2_O_2_ environment, no black dots (Mn_3_O_4_) were observed in TEM micrograph (**Figure S8A**), indicating the complete and successful decomposition of gatekeepers. As shown in **Figure S8B**, Mn_3_O_4_ nanoparticles could dissolute accelerated into Mn^2+^ ions in acidic environment with at the same H_2_O_2_ concentration. The Mn_3_O_4_ susceptibility against GSH (most abundant reducing agent exists in solid tumors) is also concerned. The findings in **Figure S9** revealed that Mn_3_O_4_ nanoparticles could also be disintegrated into Mn2+ ions by GSH trigger.

To verify the responsive and triggered release of IR780 in this gatekeeping system, the release profiles of IR780 were recorded by UV-vis-NIR absorption under an* in vitro-*simulated H_2_O_2_-rich tumor microenvironment (**Figure 2E**). To enhance the release of hydrophobic cargo, we also added a small amount of ethanol to the simulated conditions. In the absence of H_2_O_2_, less than 9% of IR780 was released after 12 h, while 35% release of photosensitizer was observed within 6 h in the noncapping condition. Upon exposing the nanocomposite to 1 mM H_2_O_2_, a rapid release behavior was observed in the first 6 h, suggesting the oxidant-responsive and controlled release of cargo under tumor- mimicking conditions. We next compared the ROS generation by Mn_3_O_4_@MSNs@IR780 and IR780 alone through the fluorescence intensity of oxidized SOSG, as SOSG oxidation led to increased fluorescence in the presence of ROS. The Mn_3_O_4_@MSNs@IR780 nanoparticles resulted in the highest accumulation of fluorescence and significantly higher fluorescence than that of IR780 alone under 808 nm laser irradiation. It is remarkable that the PDT activity of Mn_3_O_4_@MSNs@IR780 + laser was even weaker than that of the IR780 + laser group in the absence of H_2_O_2_, which provided further evidence that these nanoparticles responded only to the tumor microenvironment to release IR780, achieving superior biosafety (**Figure 2F**). These results demonstrate that these multifunctional biocompatible drug nanocarriers can respond to H_2_O_2_-rich environments, intensely increasing the oxygen concentration and successfully releasing drugs to realize mitochondrion-targeted PDT.

### Subcellular localization of Mn_3_O_4_@MSNs@IR780

**Figure 3:**
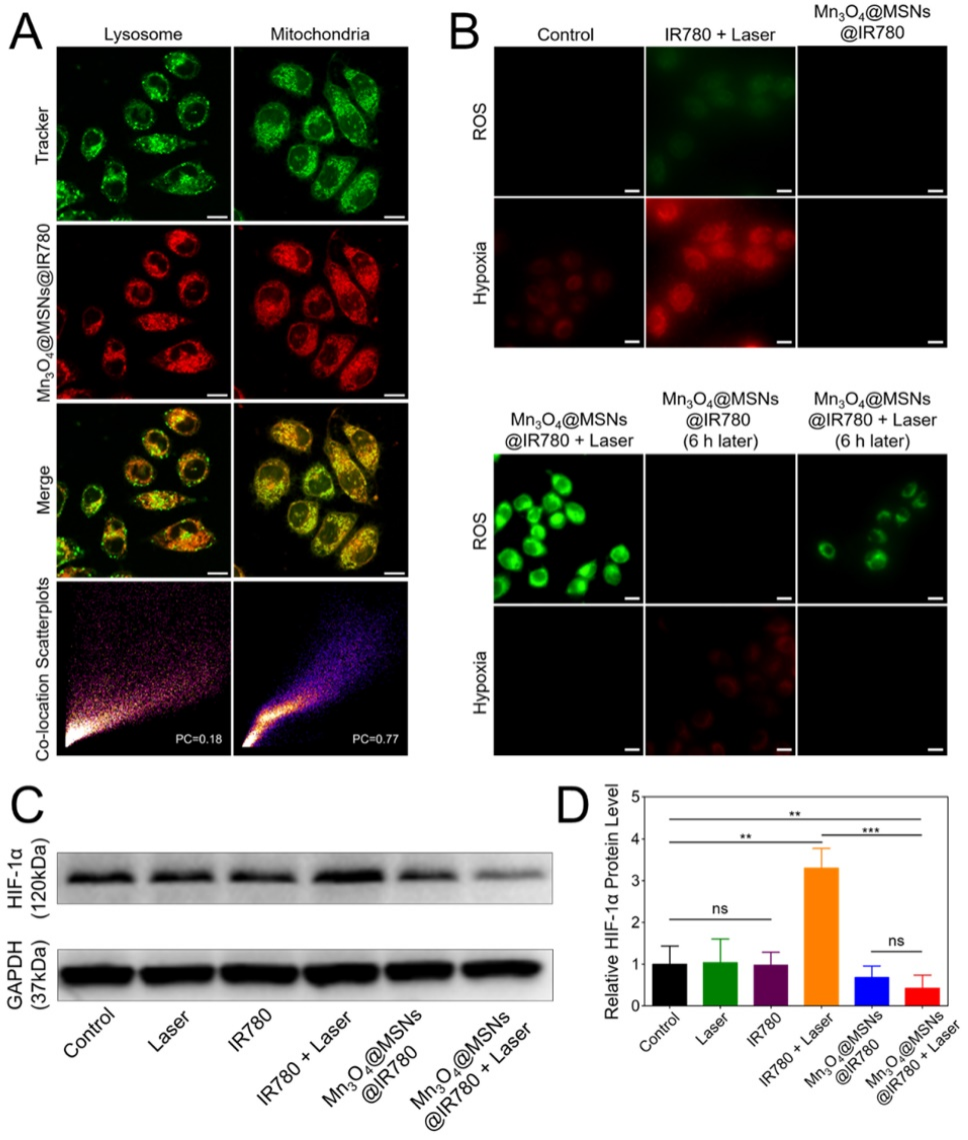
Mitochondrial targeting and inhibiting mitochondrial respiration property of Mn_3_O_4_@MSNs@IR780 nanoparticles *in vitro*. (A) Subcellular localization compared to lysosome and mitochondria trackers using CLSM. The scale bars are 10 μm. (B) CLSM images of MKN-45P cells using ROS / hypoxia detection probes as indicators. Green fluorescence indicates ROS generation and red fluorescence indicates hypoxia in cells. The scale bars are 20 μm. (C-D) Western blottings and quantitative analysis of HIF-1α protein level in cell supernatant (n = 3). Data is shown as mean ± SD. *p < 0.05; **p < 0.01 and ***p < 0.001.

As a lipophilic cation, IR780 can bind mitochondria specifically due to the higher mitochondrial membrane potential in tumor cells 37,38. To identify our conjecture that Mn_3_O_4_@MSNs@IR780 can inhibit mitochondrial respiration through mitochondrion-targeted PDT, we compared the subcellular localization of Mn_3_O_4_@MSNs@IR780 and the designated organelles *in vitro*. The red signal of Mn_3_O_4_@MSNs@IR780 showed extremely similar localization to the green fluorescence of mitochondria. Comparatively, the localization was not similar to that of the green fluorescence of lysosomes, which suggested the mitochondrion targeting of Mn_3_O_4_@MSNs@IR780 in cancer cells (**Figure 3A**). Colocalization analysis of Mn_3_O_4_@MSNs@IR780 with mitochondria tracker exhibited a similar trend while lysosome tracker performed different trend (**Figure S10**). Additionally, the subcellular localization of Mn_3_O_4_@MSNs and designated organelles is shown in **Figure S11**.

### Inhibiting mitochondrial respiration ability *in vitro*

The mitochondrion targeting ability of IR780 potentially enlarged the efficacy of PDT because mitochondria are highly susceptible to hyperthermia and ROS. After identifying the mitochondrion-targeting ability of Mn_3_O_4_@MSNs@IR780, further experiments were conducted to observe the inhibition of mitochondrial respiration *in vitro*. Green fluorescence (ROS) was detected in the IR780 + laser group and the Mn_3_O_4_@MSNs@IR780 + laser group. The control group exhibited little red fluorescence, showing the hypoxic microenvironment in cancer cells. Meanwhile, obvious red fluorescence representing hypoxia can be observed in the IR780 + laser group, indicating that IR780 will aggravate hypoxia through PDT, whereas Mn_3_O_4_@MSNs@IR780 produce ROS upon 808 nm laser irradiation without aggravating hypoxia. We also noticed that the red fluorescence disappeared after Mn_3_O_4_@MSNs@IR780 was added but reappeared after 6 h of coculture, demonstrating that although Mn_3_O_4_ can generate O_2_ in cancer cells, the inhibition of hypoxia is not sustainable. In contrast, 6 h after being treated with Mn_3_O_4_@MSNs@IR780 + laser, no red fluorescence was observed in cells (**Figure 3B**). Further flow cytometry using ROS / hypoxia detection probes pointing to the same results (**Figure S12**).

The data above confirmed our conjecture that due to the mitochondrion targeting ability, Mn_3_O_4_@MSNs@IR780 can sustainably inhibit mitochondrial respiratory function and thus inhibit the hypoxic microenvironment. Thus, the sustainable inhibition of tumor hypoxia through the PDT approach was first reported and may solve the problems that result from PDT consuming oxygen in the tumor microenvironment, which can lead to poor prognosis, such as recurrence and metastasis.

### Hypoxia detection in cancer cells

The next purpose was to examine the assumption that Mn_3_O_4_@MSNs@IR780 could inhibit hypoxia-related signaling pathways. According to western blot results, the cells in the control, laser and IR780 groups exhibited similar expression of HIF-1α with no statistical difference. However, the level of HIF-1α protein in the IR780 + laser group was much higher than in the other groups, demonstrating that PDT therapy alone may result in a more hypoxic microenvironment in cancer cells with a worse prognosis. Comparatively, HIF-1α levels in the Mn_3_O_4_@MSNs@IR780+laser group was the lowest among the six groups (**Figure 3C**). In addition, the HIF-1α protein level in the Mn_3_O_4_@MSNs@IR780 + laser group was significantly lower than that in the control group (*p = 0.0098), demonstrating that Mn_3_O_4_@MSNs@IR780 could significantly alleviate tumor hypoxia while consuming oxygen to generate ROS (**Figure 3D**). Based on the results above, we concluded that Mn_3_O_4_@MSNs@IR780 could inhibit hypoxia-related signaling pathways, thus enhancing the curative effects of PDT through oxygen generation and the sustained inhibition of mitochondrial respiration.

### ROS generation in cancer cells

**Figure 4:**
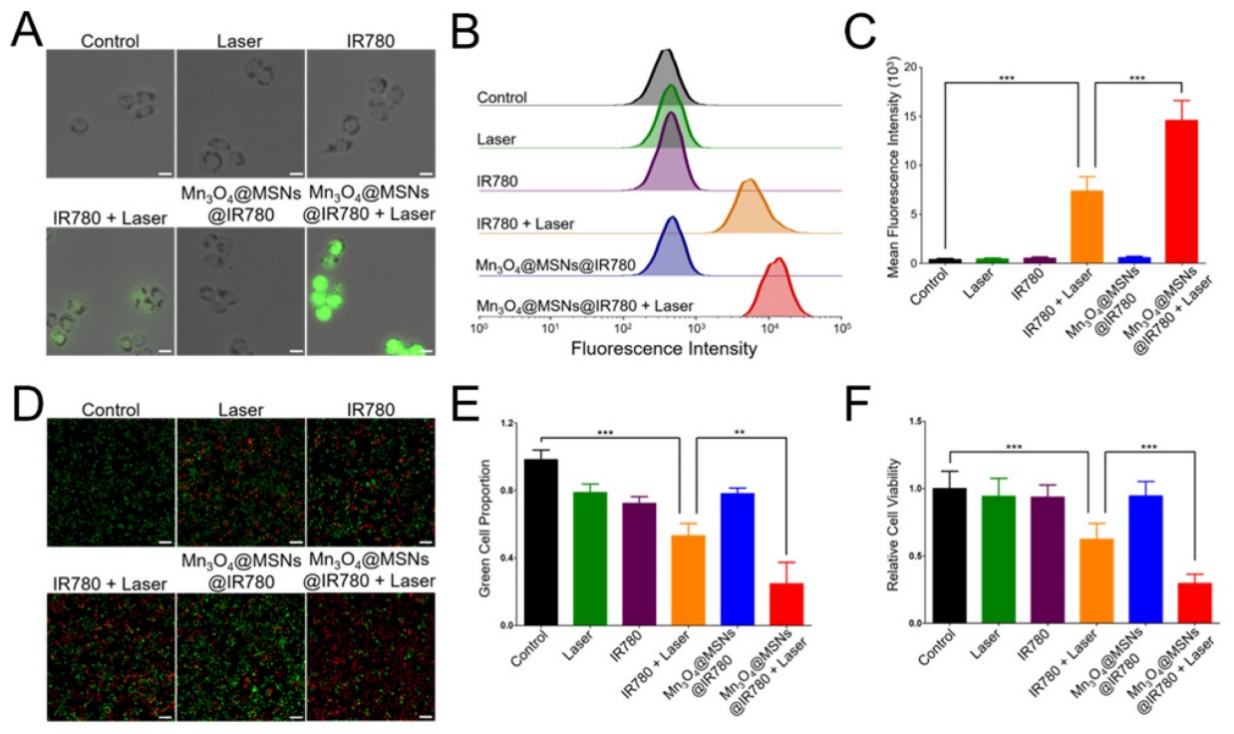
** ROS generation and cytotoxicity in MKN-45P cells after different treatments. (A)** CLSM images of ROS generation. The scale bars are 20 μm. **(B)** Flow cytometry analysis of ROS generation. **(C)** Mean fluorescence intensities of ROS generation (n = 3). **(D)** CLSM images of MKN-45P cells using CAM/PI double stain kit as indicators. Green fluorescence indicates live cells and red fluorescence indicates dead cells. The scale bars are 50 μm. **(E)** Proportion of green cells in total (green and red) cells (n = 3). **(F)** Relative cell viability using CCK-8 kit (n = 6). Data is shown as mean ± SD. *p < 0.05; **p < 0.01 and ***p < 0.001.

Because of their high cytotoxicity, ROS can kill tumor cells directly 39,40. To detect whether Mn_3_O_4_@MSNs@IR780 could generate ROS in cells upon 808 nm laser irradiation, IR780 and Mn_3_O_4_@MSNs@IR780 were incubated with MKN-45P cells, and ROS generation was detected by DCFH-DA. The Mn_3_O_4_@MSNs@IR780 + laser group showed high green fluorescence under an 808 nm laser (1 W·cm^-2^, 5 min), suggesting ROS generation (**Figure 4A**). In contrast, low green fluorescence was observed in cells in the IR780 + laser group, demonstrating that Mn_3_O_4_@MSNs@IR780 could enhance the PDT effect. The ROS production ability was further quantitatively analyzed using flow cytometry (**Figure 4B-C**). Cells in the Mn_3_O_4_@MSNs@IR780 + laser group exhibited the highest fluorescence intensity, which was much higher than that of the IR780 + laser group, while the other groups exhibited negligible fluorescence (IR780 + laser vs Mn_3_O_4_@MSNs@IR780 + laser, ***p < 0.001). These results confirmed that adequate amounts of ROS could be selectively produced in cancer cells through Mn_3_O_4_@MSNs@IR780 and NIR irradiation, which meant that Mn_3_O_4_@MSNs contributed significantly to enhancing the PDT outcome of IR780.

### *In vitro* therapy against cancer cells

After identifying the PDT effect and mitochondrion-targeting ability of Mn_3_O_4_@MSNs@IR780, CAM/PI and the CCK-8 protocol were used to evaluate the cytotoxicity against MKN-45P cells. No obvious red cells were observed in the control group, while a small number of red cells were observed in the laser, IR780 and Mn_3_O_4_@MSNs@IR780 groups (**Figure 4D**). Meanwhile, a moderate number of MKN-45P cells were observed in red color after incubation with IR780 and exposure to 808 nm laser irradiation, suggesting less powerful damage against the cancer cells. In the Mn_3_O_4_@MSNs@IR780 + laser group, almost all MKN-45P cells emitted red fluorescence with no visible green cells. The green cell proportion in these various groups pointed to the same results (**Figure 4E**). Meanwhile, CCK-8 was used to further detect the *in vitro* curative effects of Mn_3_O_4_@MSNs@IR780 upon 808 nm laser irradiation. There were no obvious significant differences in cell viability among the control, laser, IR780 and Mn_3_O_4_@MSNs@IR780 groups (**Figure 4F**).

Nevertheless, the cytotoxicity of the Mn_3_O_4_@MSNs@IR780 + laser group was significantly higher than that of the IR780 + laser group when the IR780 concentration was fixed (5 µg·mL^-1^). These results indicate that Mn_3_O_4_@MSNs@IR780 exhibited superior biosafety property *in vitro* and had a powerful phototherapeutic effect against MKN-45P cells under 808 nm laser irradiation, which could be further used to cure tumors.

### *In vivo* NIR fluorescence imaging and biodistribution

**Figure 5:**
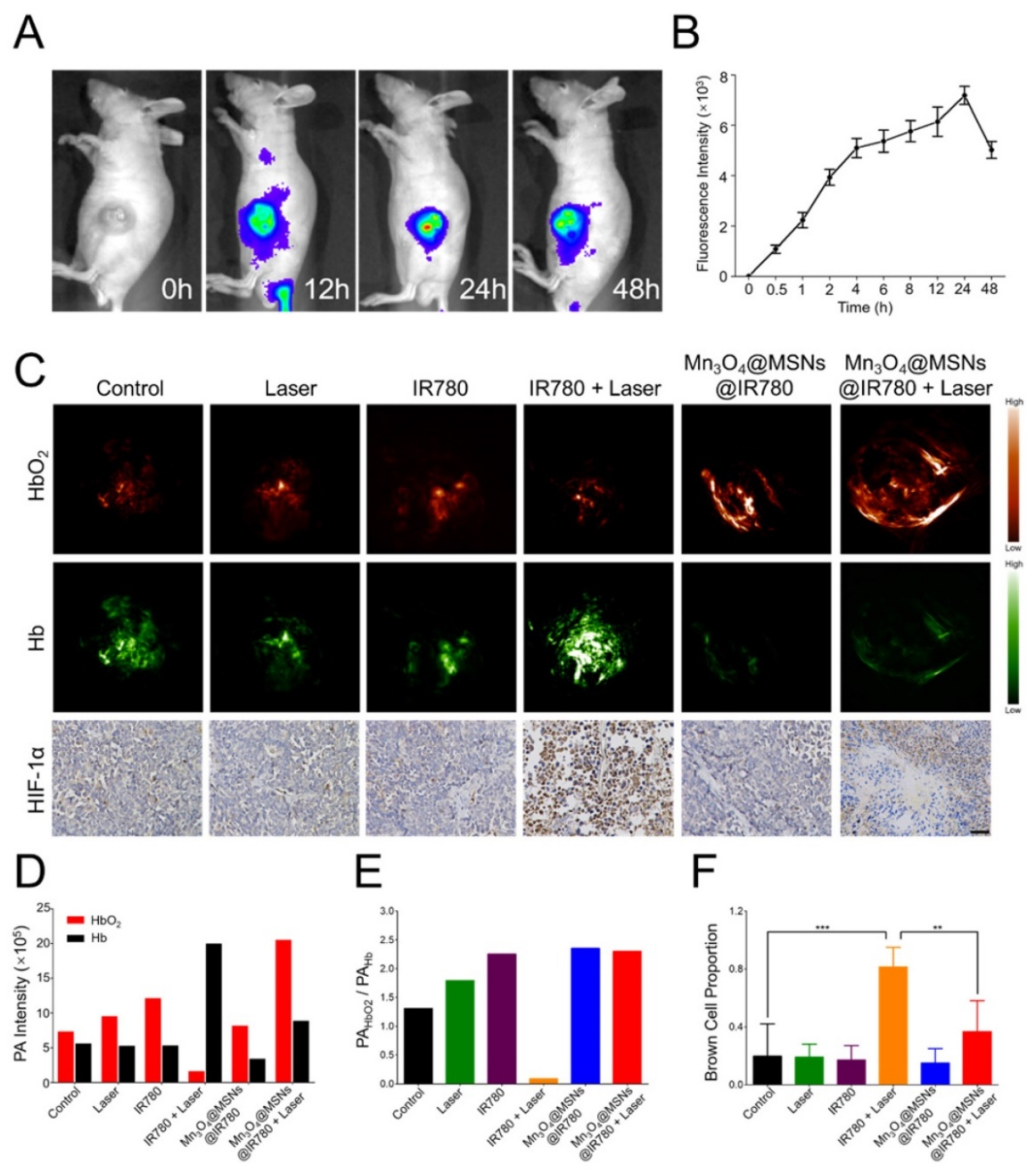
Biodistribution of Mn_3_O_4_@MSNs@IR780 *in vivo* and detection of tumor hypoxia conditions after different treatments. (A) *In vivo* real-time NIR fluorescence images of MKN-45P xenografts after injection of Mn_3_O_4_@MSNs@IR780 at different time points. (B) *In vivo* fluorescence signal intensity of tumor area after injection of Mn_3_O_4_@MSNs@IR780 at different time (n = 4). (C) PA images and HIF-1α staining of tumor tissues 24h after injection. HbO_2_ (λ = 850 nm) and Hb (λ = 700 nm) monitor the real-time tumor oxygenation. The scale bars are 50 μm. Blue indicate HIF-1α negative cells while brown indicate HIF-1α positive cells. (D) PA intensity of HbO_2_ and Hb 24h after injection. (E) The ratio of HbO_2_ PA intensity to Hb PA intensity. (F) Proportion of brown cells in total (blue and brown) cells (n = 4). Data is shown as mean ± SD. *p < 0.05; **p < 0.01 and ***p < 0.001.

After *in vitro* studies, MKN-45P tumor xenograft models were used for *in vivo* studies. Our first goal was to determine the appropriate irradiation time point after administration. The tumor accumulation and biodistribution of Mn_3_O_4_@MSNs@IR780 were then monitored through the fluorescent property of IR780 with NIR fluorescence imaging performance (λ_ex_ / λ_em_ = 745 nm / 820 nm). After tail vein injection, real-time NIR images were acquired at different times (**Figure 5A**). Different colors were used to display different fluorescence intensities, which decreased in the order of red, yellow and blue. The fluorescence signal began to accumulate at the tumor 0.5 h post injection and could be observed in tumor areas 4 h after injection, while the strongest fluorescence signal was detected in tumor tissues 24 h after injection (**Figure 5B**), indicating the effective accumulation of Mn_3_O_4_@MSNs@IR780 in tumor tissues through the enhanced permeability and retention (EPR) effect. According to the ICP results of manganese content, Mn_3_O_4_@MSNs@IR780 accumulated the most in liver tissues. Additionally, manganese concentration in tumor tissues reach the peak 24 h after injection, showing the same results as *in vivo* NIR fluorescence imaging (**Figure S13).**

### *In vivo* monitoring of hypoxic tumor conditions

To further confirm whether Mn_3_O_4_@MSNs@IR780 can inhibit hypoxia in the tumor area, hypoxic conditions in tumor tissues were detected using PA imaging to measure the dynamics of hypoxia (Hb, 700 nm) and oxygen (HbO_2_, 850 nm) in tumor tissues after different treatments (**Figure 5C**). The highest PA signal of HbO_2_ and the lowest of Hb were observed in the Mn_3_O_4_@MSNs@IR780 + laser group 24 h after injection. Interestingly, the highest PA signal of Hb and the lowest of HbO_2_ were observed in the IR780+laser group 24 h after injection. There were no obvious differences among the control, laser, IR780, and Mn_3_O_4_@MSNs@IR780 groups (**Figure 5D-E**). These results indicated that Mn_3_O_4_@MSNs@IR780 can prevent tumor hypoxia by increasing the local oxygen supply.

Afterwards, tumor tissues were harvested for HIF-1α staining to determine whether the hypoxia-related signaling pathways were inhibited (**Figure 5F**). The IR780 + laser group exhibited a higher HIF-1α level than that in the control, laser, IR780 and Mn_3_O_4_@MSNs@IR780 groups, while the Mn_3_O_4_@MSNs@IR780 + laser group showed no detectable elevation in HIF-1α levels compared to the control group (IR780 + laser vs Mn_3_O_4_@MSNs@IR780 + laser, **p < 0.01), suggesting that the treatment can effectively ameliorate the hypoxic microenvironment while inhibiting the hypoxia signaling pathway of cancer cells.

### *In vivo* antitumor therapy

**Figure 6:**
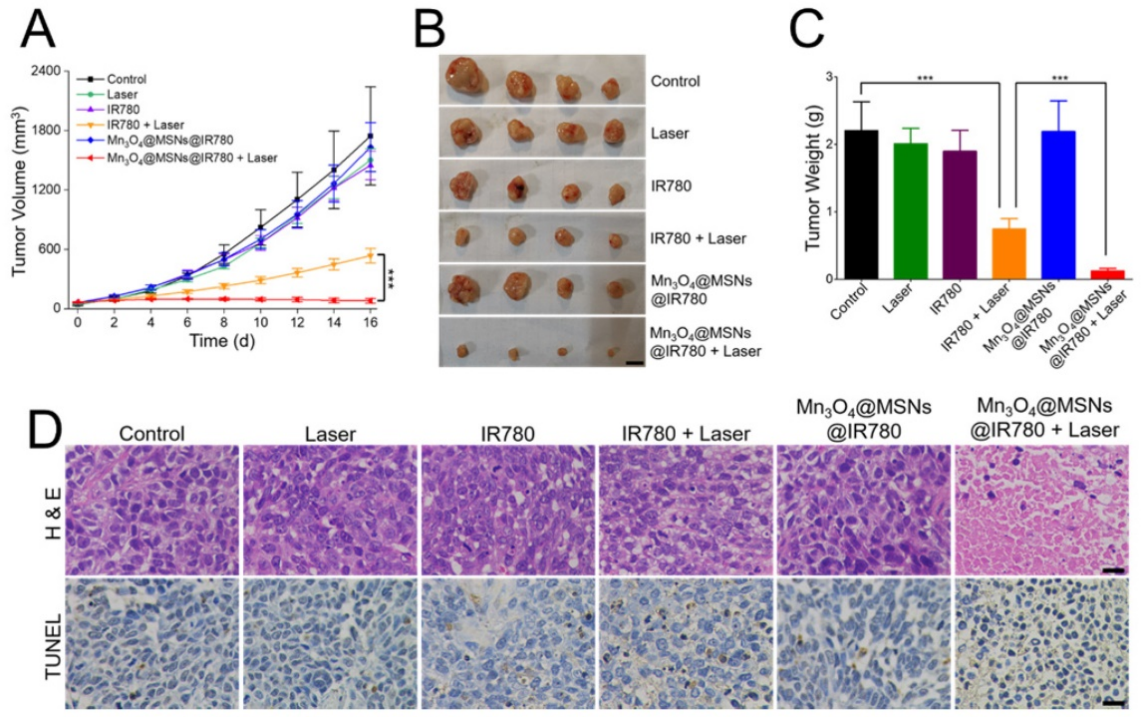
Detection of the *in vivo* anti-tumor effect after different treatments (n = 4). (A) Tumor growth curves. (B-C) Photograph and weight of tumors. The scale bars are 1 cm. (D) H & E staining and TUNEL staining tumor sections. The scale bars are 50 μm. Data is shown as mean ± SD. *p < 0.05; **p < 0.01 and ***p < 0.001.

After indicating that Mn_3_O_4_@MSNs@IR780 could efficiently kill MKN-45P cells *in vitro* and ameliorate the hypoxic microenvironment *in vivo* upon 808 nm laser irradiation, the *in vivo* antitumor effect was further examined. According to the *in vivo* NIR imaging and biodistribution results, 24 h was selected as the time point for 808 nm laser irradiation after one i.v. injection (1 W·cm^-2^, 5 min). The final IR780 concentration was 25 µg·kg^-1^ per mouse. The control group showed rapid tumor growth with almost no therapeutic effects, while the laser, IR780 and Mn_3_O_4_@MSNs@IR780 groups showed similar trends to the control group (**Figure 6A**). For mice in the IR780 + laser group, the tumors grew slowly in the first four days, but unfortunately, they grew rapidly after four days. Thus, we conjectured that the unsustainable inhibition of tumor hypoxia might limit the curative effect and lead to tumor recurrence and poor prognosis. In contrast, almost no increase in tumor volume was observed in the Mn_3_O_4_@MSNs@IR780 + laser group, indicating a strikingly enhanced therapeutic outcome (IR780 + laser vs Mn_3_O_4_@MSNs@IR780 + laser, ***p < 0.001). Additionally, the photographs and weights of tumors exhibited almost identical trends to the changes in tumor volume (**Figure 6B-C**). Combined with the *in vitro* results, we concluded that Mn_3_O_4_@MSNs@IR780 could greatly enhance the PDT curative effects to inhibit tumor growth and recurrence mainly because of the enhanced PDT efficacy and inhibition of hypoxia recovery.

### Biosafety analysis

**Figure 7:**
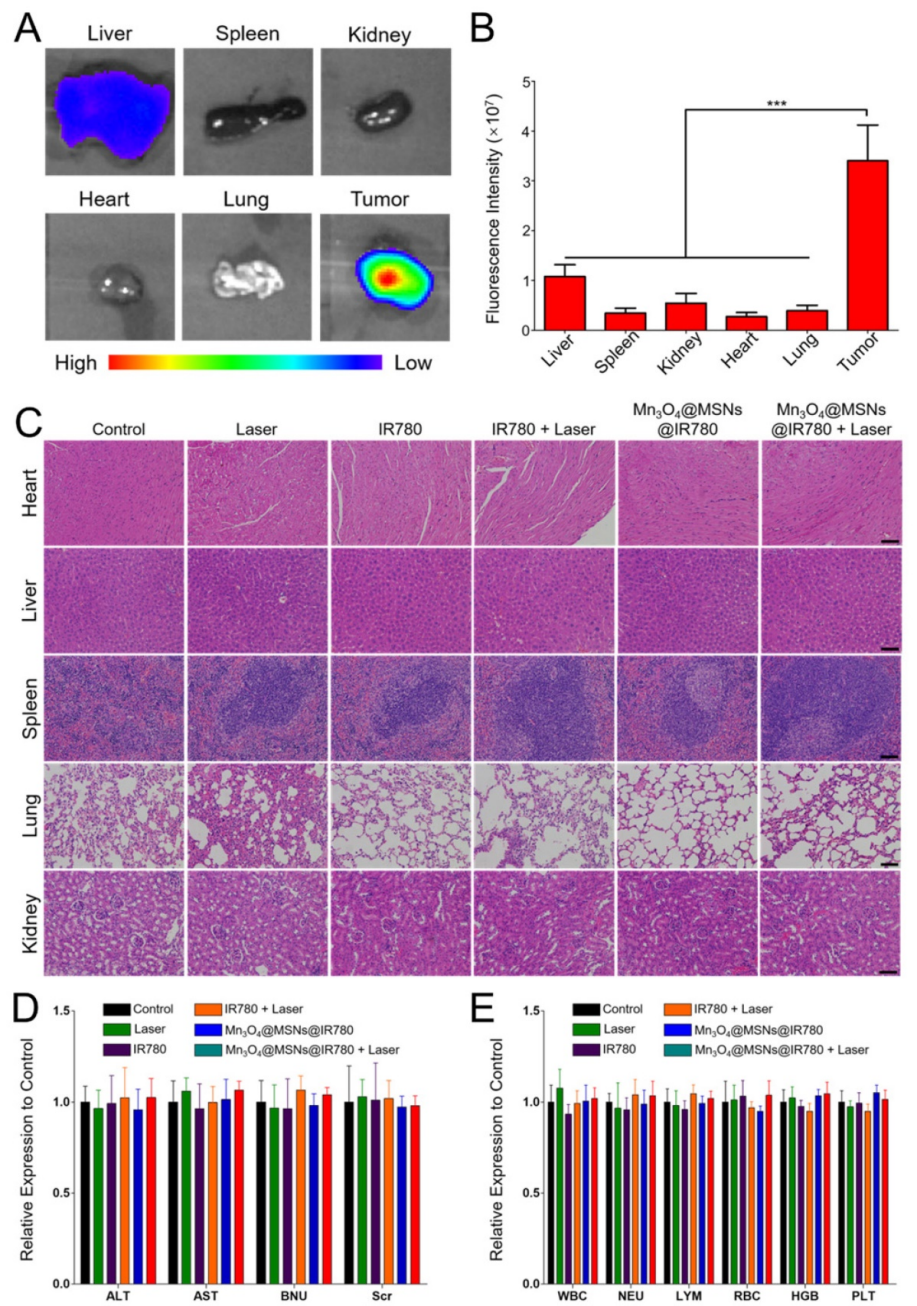
Biosafety Analysis of different treatments. (A) *Ex vivo* NIR fluorescence of major organs and tumor tissue 24h after injection of Mn_3_O_4_@MSNs@IR780. (B) Fluorescence signal intensity of major organs and tumor tissue (n = 4). (C) H & E staining major organs sections. The scale bars are 50 μm. (D) Hematology assay of white blood cell (WBC), neutrophils (NEU), lymphocytes (LYM), red blood cell (RBC), hemoglobin (HGB) and platelets (PLT) levels (n = 4). (E) Serum biochemical study of alanine aminotransferase (ALT), aspartate aminotransferase (AST), blood urea nitrogen (BUN) and serum creatinine (Scr) levels after different treatments (n = 4). Data is shown as mean ± SD. *p < 0.05; **p < 0.01 and ***p < 0.001.

To evaluate the *in vivo* biosafety of Mn_3_O_4_@MSNs@IR780, NIR fluorescence imaging of main organs and tumor tissues was acquired 24 h after injection (**Figure 7A-B**). The fluorescent intensity in the tumor (3.4 × 10^7^) was significantly higher than the second high fluorescent intensity in the liver (1.08 × 10^7^), indicating that Mn_3_O_4_@MSNs@IR780 accumulated most in the tumor (***p < 0.001). Next, major organs, including the heart, liver, spleen, lung and kidney, were collected at the end of different treatments. All groups exhibited negligible inflammation lesions, histological abnormalities and necrosis, which strongly indicated the good biocompatibility of Mn_3_O_4_@MSNs@IR780 (**Figure 7C**). In addition, almost no weight fluctuations in the mice were observed during the therapy (**Figure S14**).

Finally, hematological and biochemical assays were performed to evaluate the potential cytotoxicity of Mn_3_O_4_@MSNs@IR780 after photodynamic therapy (**Figure 7D-E**). No significant differences were observed in immune response (WBC, NEU and LYM), cytotoxicity (RBC and HGB), spleen function (PLT), liver function (ALT and AST) and renal function (BUN and Scr) at the end of treatments in all groups compared with the control group. These results verified the high therapeutic biosafety of Mn_3_O_4_@MSNs@IR780 mainly because of the tumor-targeted drug delivery and treatment, which reduced the side effects on non-tumor organs.

## Conclusions

In this study, a Mn_3_O_4_@MSNs@IR780 nanocomposite was successfully prepared to scavenge the tumor hypoxic microenvironment by self-generating oxygen and the mitochondrion-targeted destruction of respiration in cancer cells, further enhancing PDT efficiency and therapeutic outcome. *In vitro* studies indicated that Mn_3_O_4_ nanoparticles could decompose H_2_O_2_ and sustainably generate oxygen under H_2_O_2_-rich physiological conditions. During H_2_O_2_ decomposition, Mn_3_O_4_ nanoparticles were also designed to leave/dissolve from the MSN surface and thereby enable the release of inner IR780. Afterwards, the released IR780 further specifically targeted the mitochondria and generated ROS to damage their biological function, thus inhibiting respiration in cancer cells. The generation of oxygen and inhibition of cell respiration could alleviate the hypoxic tumor microenvironment, as revealed by ROS/hypoxia imaging* in vitro* and PA imaging *in vivo*. The scavenging of tumor hypoxia has also been proven to enhance PDT effects and prevent tumor recurrence, thus leading to a favorable prognosis. In conclusion, this study demonstrated a new approach for sustainably overcoming the hypoxia limitation in traditional PDT by targeted oxygen supplementation and mitochondrial destruction.

## Supplementary Material

Supplementary figures and tables.

## Abbreviations

PDT: photodynamic therapy
MSN: mesoporous silica nanoparticle
ROS: reactive oxygen species
DMF: N,N-Dimethylformamide
APTES: 3-aminopropyltriethoxysilane
CTAB: cetyltrimethylammonium bromide
TEOS: tetraethyl orthosilicate
DMSO: dimethyl sulfoxide
EDC·HCl: 1-(3-(dimethylamino)propyl)-3-ethylcarbodiimide hydrochloride
H_2_O_2_: Hydrogen Peroxide
SOSG: Singlet Oxygen Sensor Green
TEM: transmission electron microscope
SEM: scanning electron microscope
PDI: polymer dispersity index
XRD: X-ray diffraction
FTIR: Fourier transform infrared
XPS: X-ray photoelectron spectroscopy
CLSM: confocal laser scanning microscope
HIF-1α: hypoxia inducible factors-1α
CCK-8: Cell Counting Kit-8
SCID: severe combined immunodeficiency
IACUC: Institutional Animal Care and Use Committee
PA: photoacoustic
HbO_2_: Oxygenated hemoglobin
Hb: hemoglobin
H & E: hematoxylin and eosin
WBC: white blood cell
NEU: neutrophils
LYM: lymphocytes
RBC: red blood cell
HGB: hemoglobin
PLT: platelets
ALT: alanine aminotransferase
AST: aspartate aminotransferase
BUN: blood urea nitrogen
Scr: serum creatinine
SD: standard deviation
