# Supplementary material for: Self-generating oxygen enhanced mitochondrion-targeted photodynamic therapy for tumor treatment with hypoxia scavenging
Source: Theranostics. 2019 Sep 20;9(23):6809–23. doi: 10.7150/thno.36988 (PMC6815961; doi:10.7150/thno.36988)
Supplement: Supplementary file 1 — Supplementary figures and tables. [file thnov09p6809s1.pdf]

Supporting Information

Self-generating oxygen enhanced mitochondrion-targeted photodynamic therapy for tumor treatment with hypoxia scavenging

Zhengyang Yang[1,*], Jiafeng Wang[1,*], Shichao Ai[1,*], Jianfei Sun[#,2], Xiaoli Mai[#,3], and Wenxian Guan[#,1]

Affiliations:

1. Department of General Surgery, Nanjing Drum Tower Hospital, The Affiliated Hospital of Nanjing University Medical School, No. 321 Zhongshan Road, Nanjing, 210008, China.

2. School of Biological Science and Medical Engineering, Southeast University, No. 87 Dingjiaqiao, Nanjing, 210009, China

3. Department of Radiology, Nanjing Drum Tower Hospital, The Affiliated Hospital of Nanjing University Medical School, No. 321 Zhongshan Road, Nanjing, 210008, China.

*These authors contributed equally to this work.

*** Corresponding authors:**

Wenxian Guan, Ph.D.

Email: dg1835105@smail.nju.edu.cn

Tel: +86 15850502391

Address: No. 321 Zhongshan Road, Nanjing, China.

Jianfei Sun, Ph.D.

Email: sunzaghi@seu.edu.cn

Address: No. 87 Dingjiaqiao, Nanjing, 210009, China

Xiaoli Mai, Ph.D

Email: m_mxl@163.com

Address: No. 321 Zhongshan Road, Nanjing, China.

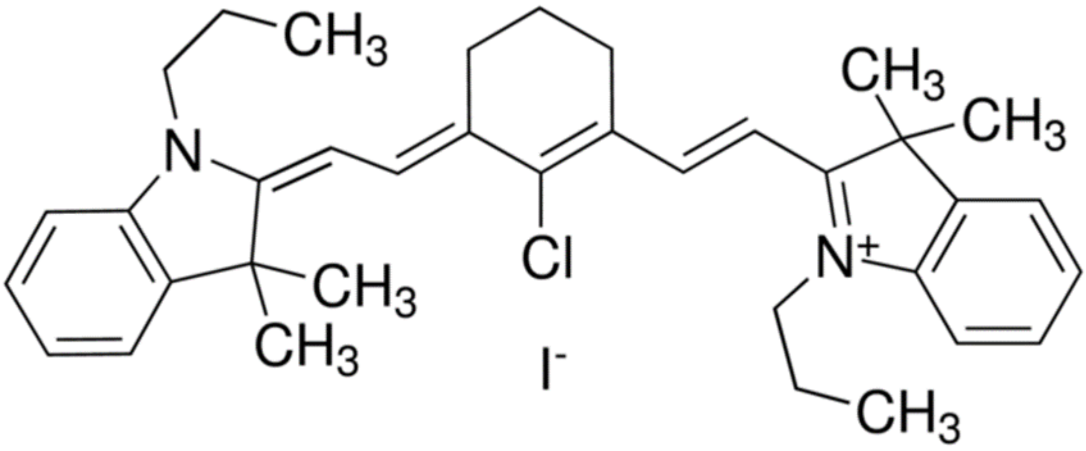

Fig. S1 Molecular structure of IR780.

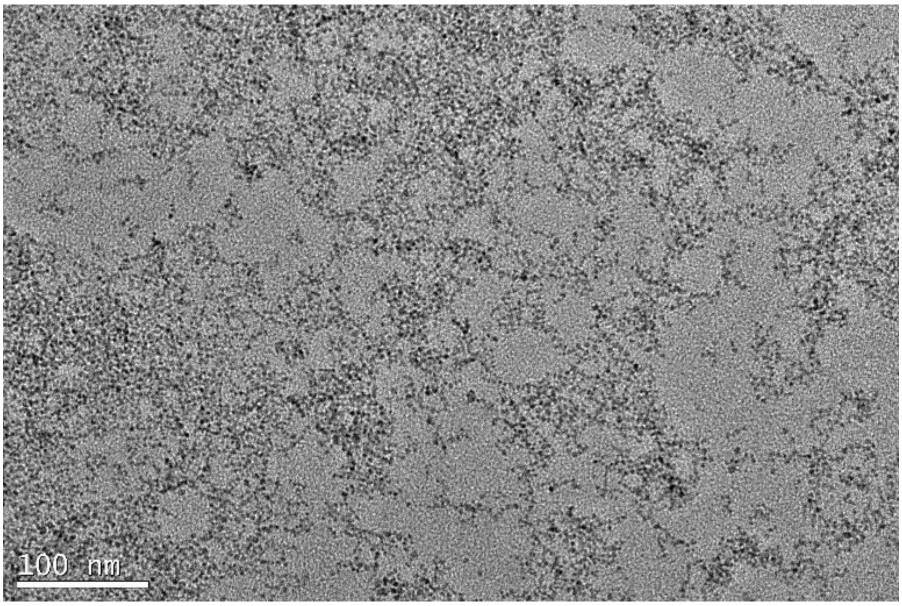

Fig. S2 TEM image of Mn_3O_4 nanoparticles.

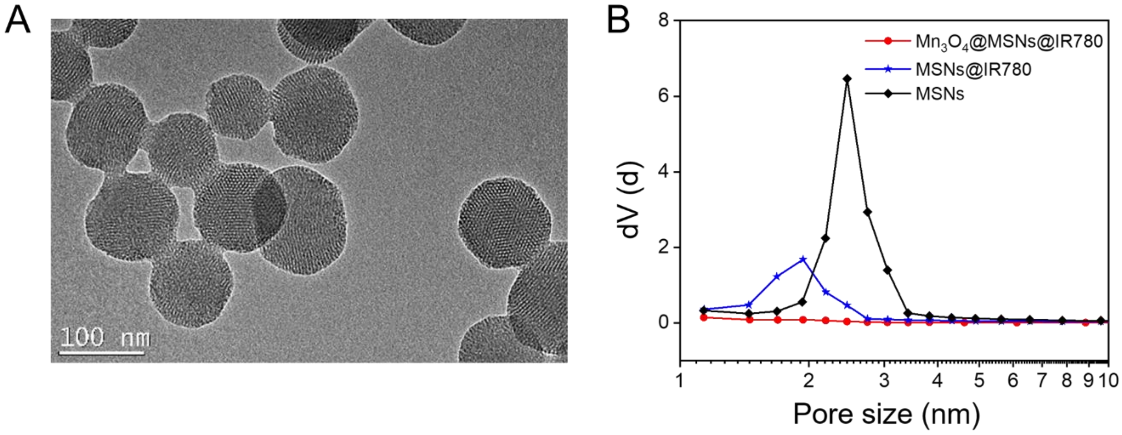

Fig. S3 (A) High resolution TEM micrograph of MSNs. (B) Corresponding pore size distributions of MSNs, MSNs@IR780, and Mn_3O_4@MSNs@IR780.

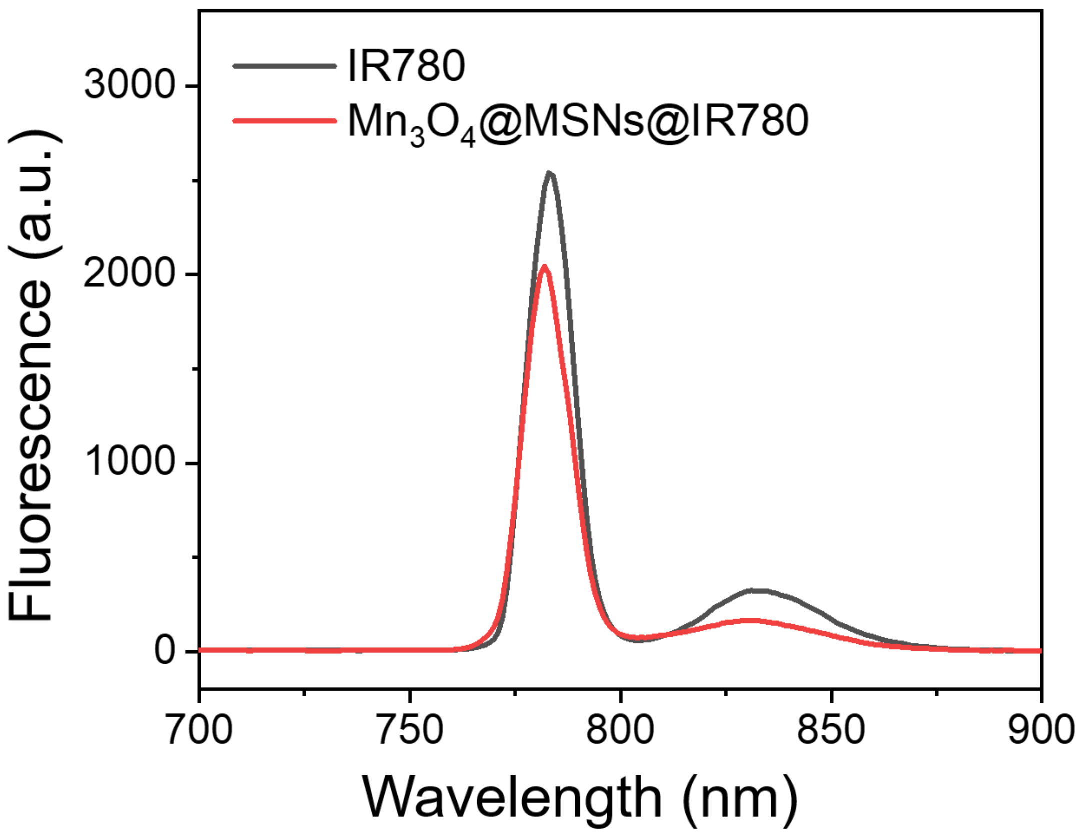

Fig. S4 Fluorescence spectrum of IR780 and Mn_3O_4@MSNs@IR780 nanoparticles.

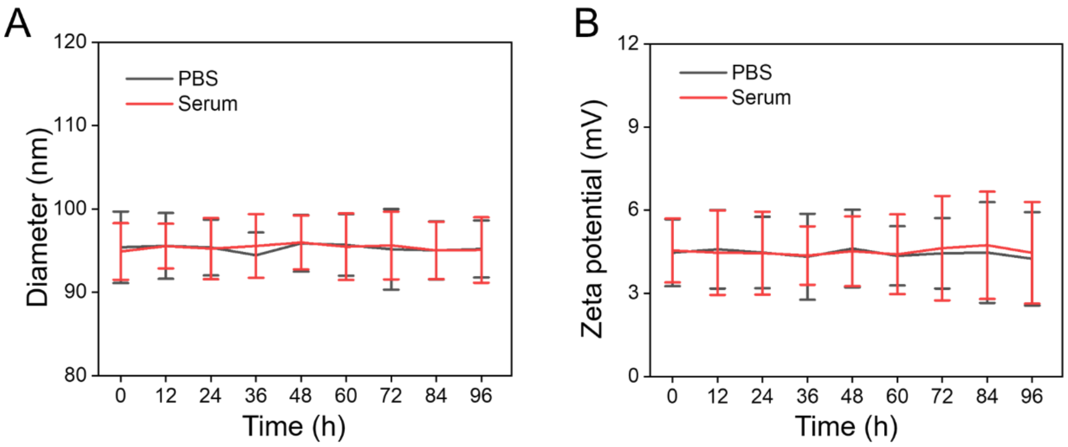

Fig. S5 (A) DLS of Mn_3O_4@MSNs@IR780 nanoparticles with time in PBS and serum every 12 h. (B) Zeta potential of Mn_3O_4@MSNs@IR780 nanoparticles with time in PBS and serum every 12 h. Data is shown as mean ± SD.

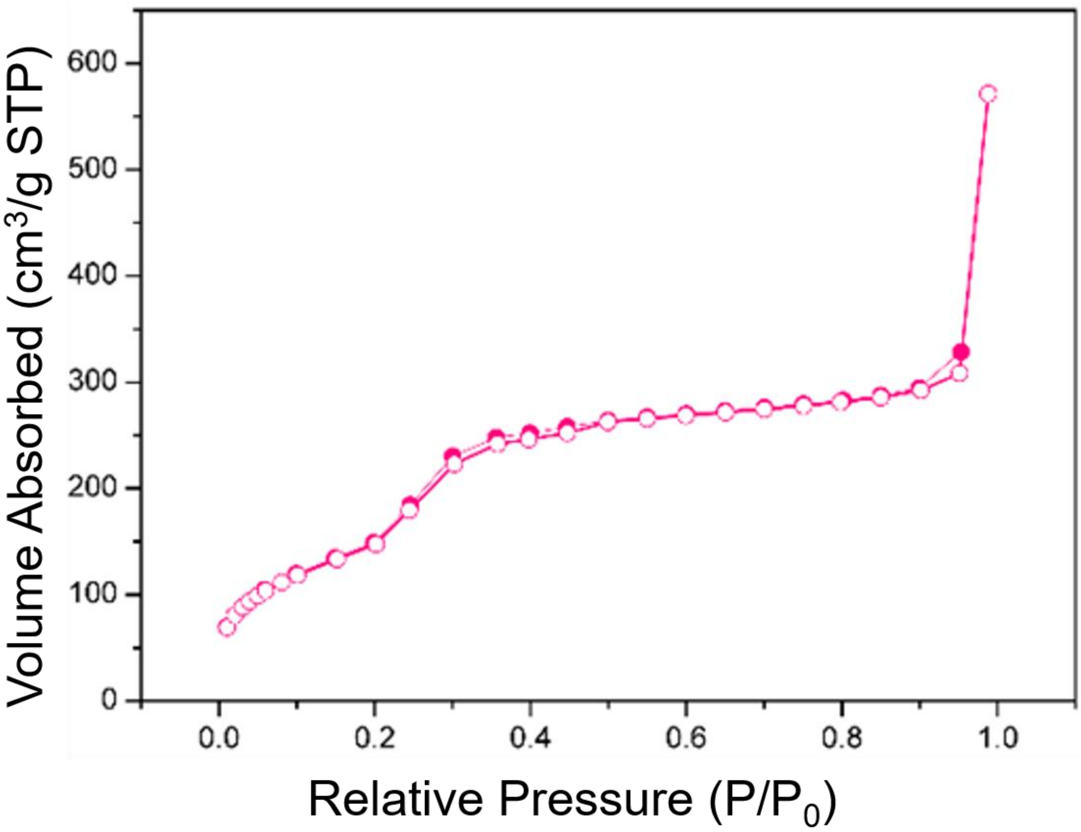

Fig. S6 Nitrogen adsorption-desorption isotherms of Mn_3O_4@MSNs@IR780 nanoparticles incubated in 1 mM H_2O_2 after 24 h.

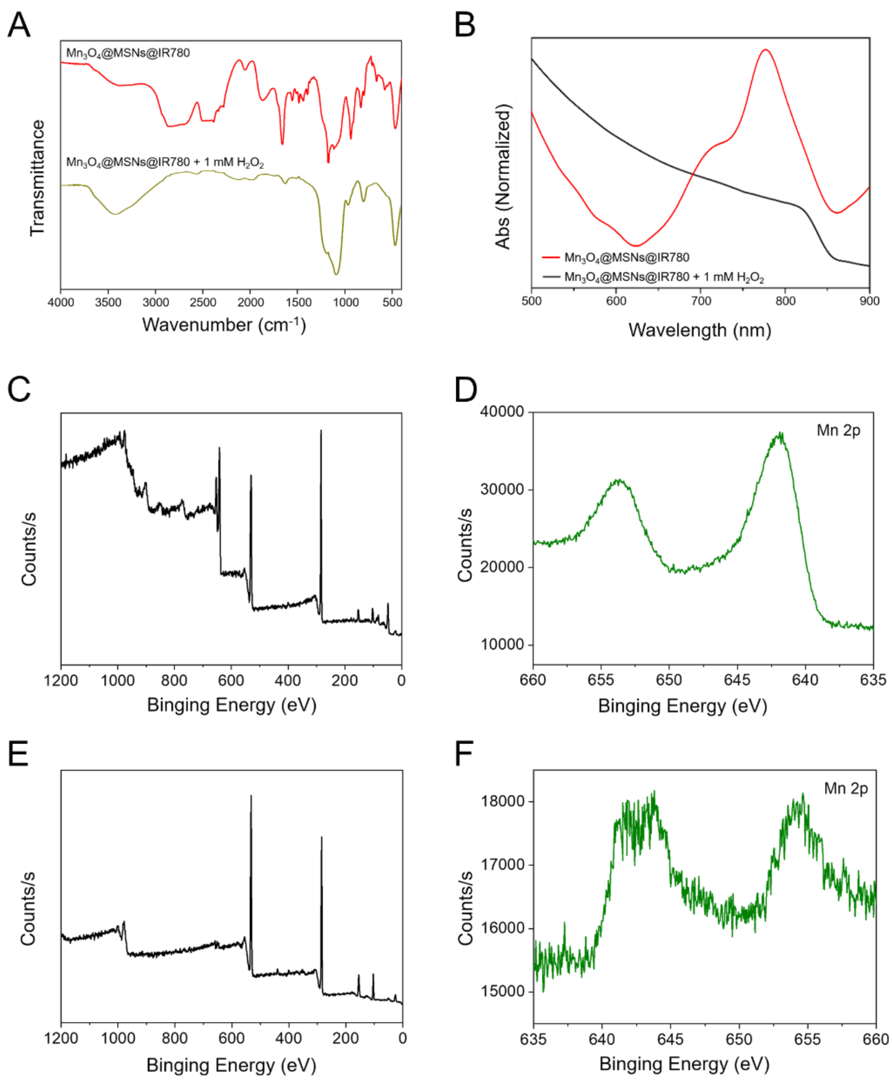

Fig. S7 (A) FTIR spectrums of Mn_3O_4@MSNs@IR780 nanoparticles before and after incubated in 1 mM H_2O_2 for 24 h. (B) UV-vis-NIR spectrums of Mn_3O_4@MSNs@IR780 nanoparticles before and after incubated in 1 mM H_2O_2 for 24 h. (C) Full survey XPS spectrum of Mn_3O_4@MSNs@IR780 nanoparticles. (D) Mn 2p peak of XPS spectrum of Mn_3O_4@MSNs@IR780 nanoparticles. (E) Full survey XPS spectrum of Mn_3O_4@MSNs@IR780 nanoparticles after incubated in 1 mM H_2O_2 for 24 h. (F) Mn 2p peak of XPS spectrum of Mn_3O_4@MSNs@IR780 nanoparticles after incubated in 1 mM H_2O_2 for 24 h.

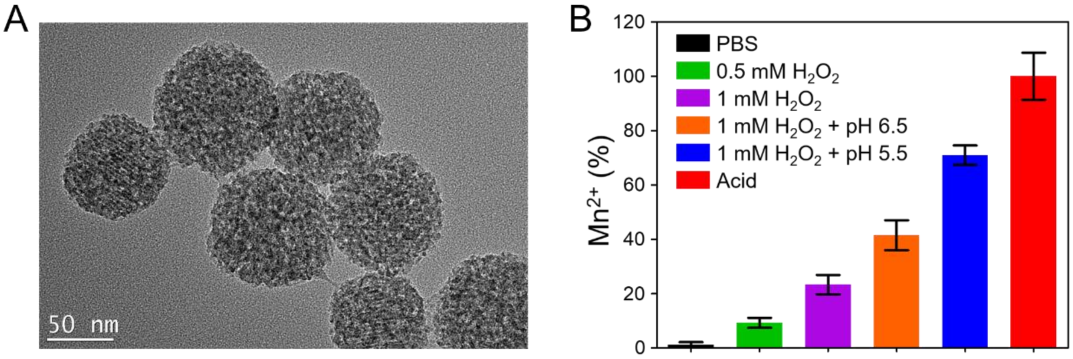

Fig. S8 (A) TEM image of Mn_3O_4@MSNs@IR780 nanoparticles incubated in 1 mM H_2O_2 (pH 5.5) after for 24 h. (B) Manganese (Mn^{2+}) percentage concentration determined by ICP analysis after subjecting to PBS, 0.5 Mm H_2O_2, 1 mM H_2O_2 at various pH and acid solution. Data is shown as mean ± SD.

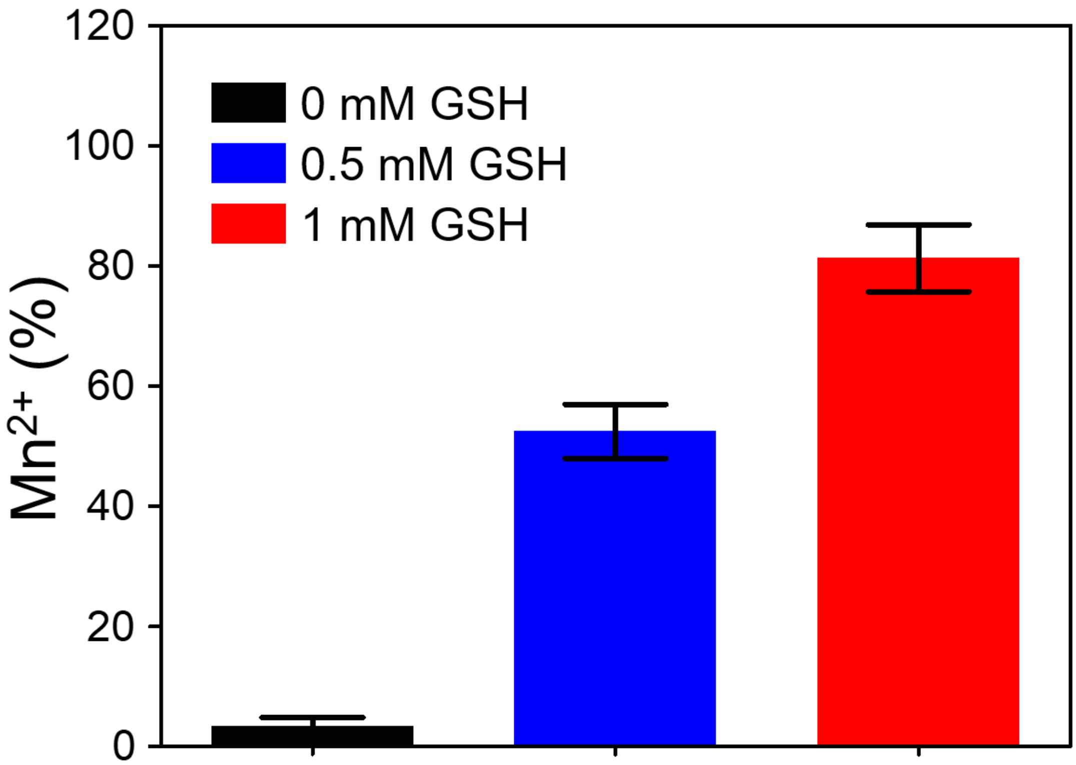

Fig. S9 Manganese (Mn^{2+}) percentage concentration determined by ICP analysis after subjecting to various GSH solutions. Data is shown as mean ± SD.

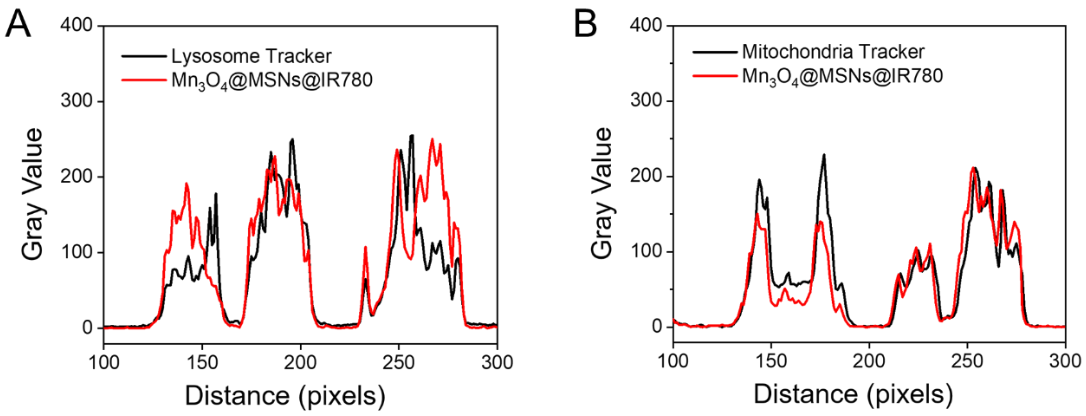

Fig. S10 (A) Colocalization analysis of Mn_3O_4@MSNs@IR780 nanoparticles in MKN45 cells with lysosome tracker. (B) Colocalization analysis of Mn_3O_4@MSNs@IR780 nanoparticles in MKN45 cells with mitochondria tracker.

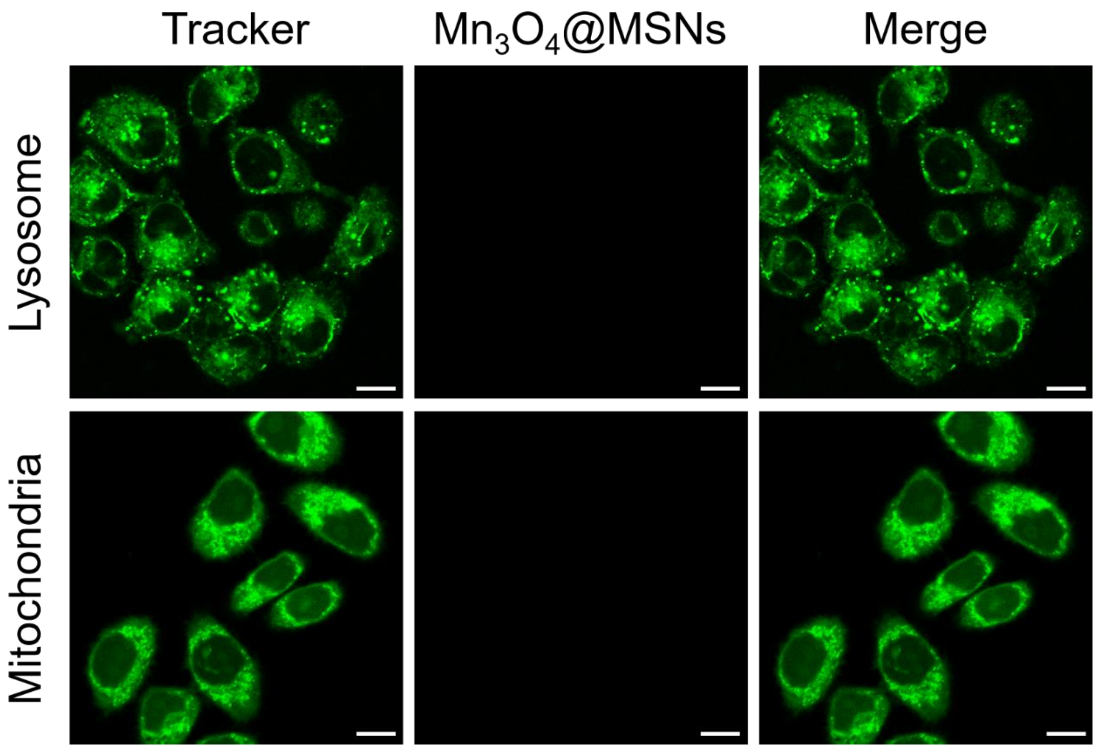

Fig. S11 Subcellular localization of Mn_3O_4@MSNs compared to lysosome and mitochondria trackers using CLSM. The scale bars are 10 μm.

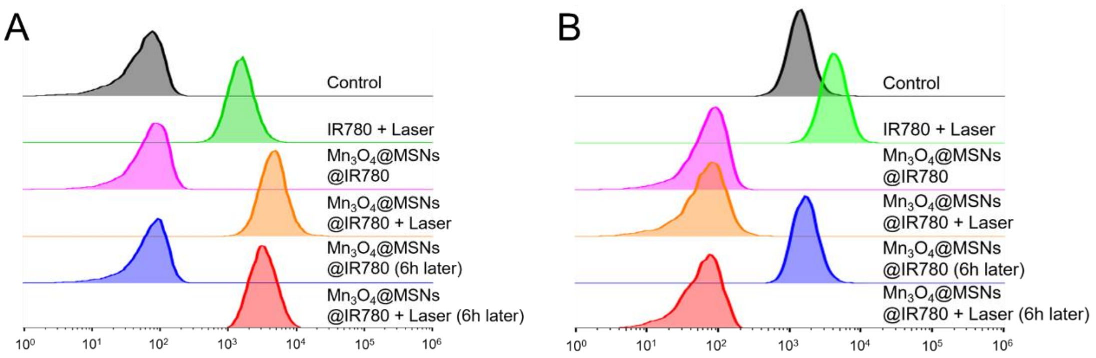

Fig. S12 Flow cytometry analysis of MKN-45P cells using ROS / hypoxia detection probes as indicators. (A) Flow cytometry analysis of ROS generation under different situations. (B) Flow cytometry analysis of hypoxia in cells under different situations.

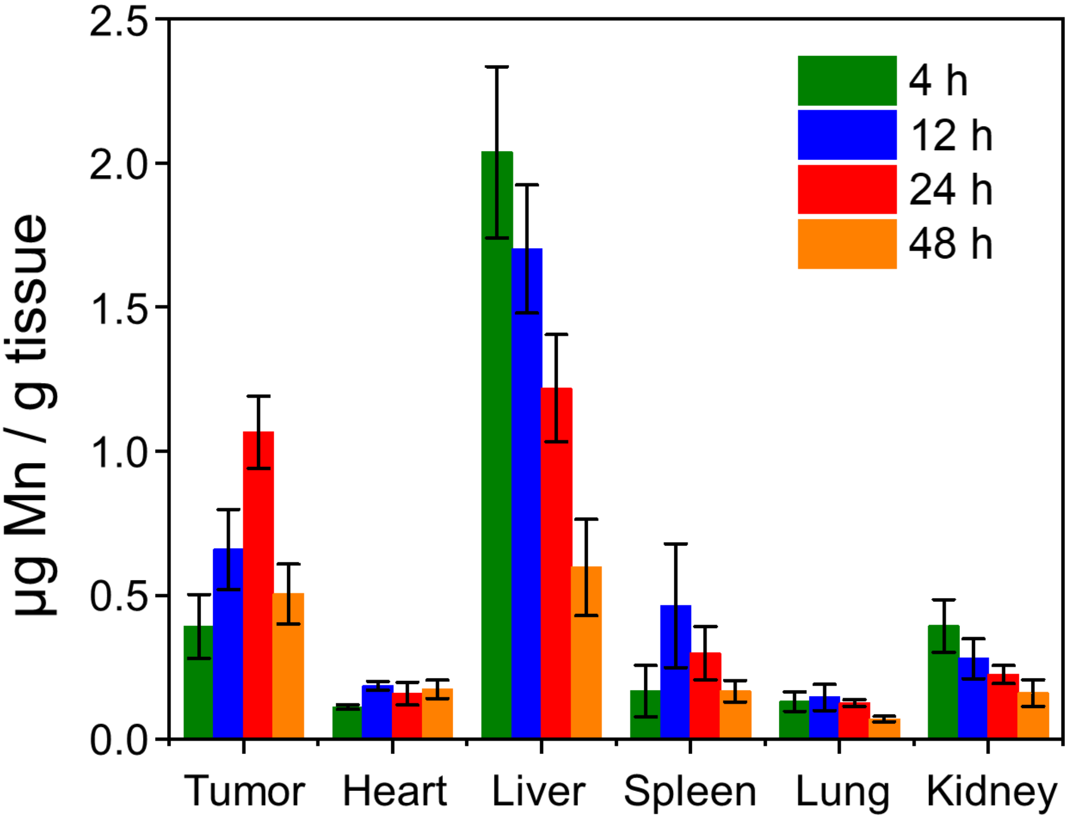

Fig. S13 In vivo biodistribution of Mn_3O_4@MSNs@IR780 nanoparticles at different time points after injection. Data is shown as mean ± SD.

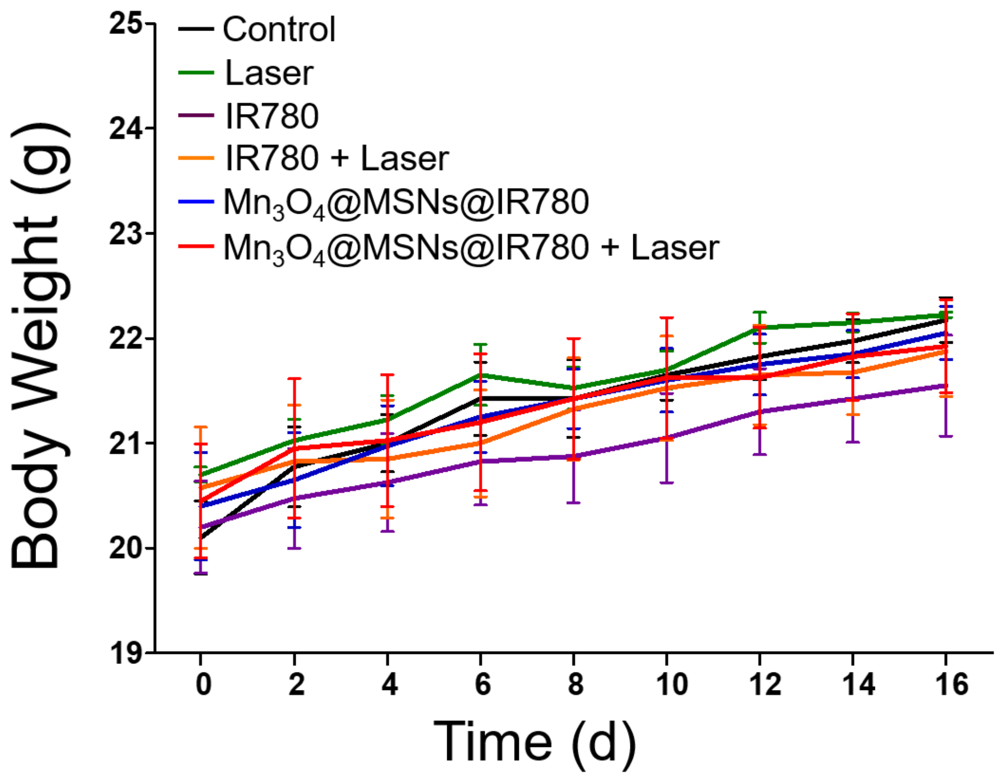

Fig. S14 Body-weight curve of six groups after various treatments (n = 4). Data is shown as mean ± SD.
